# Low Expression of SLC7A11 Confers Drug Resistance and Worse Survival in Ovarian Cancer *via* Inhibition of Cell Autophagy as a Competing Endogenous RNA

**DOI:** 10.3389/fonc.2021.744940

**Published:** 2021-11-01

**Authors:** Yao Ke, Xiaoying Chen, Yuting Su, Cuilan Chen, Shunmei Lei, Lianping Xia, Dan Wei, Han Zhang, Caihua Dong, Xia Liu, Fuqiang Yin

**Affiliations:** ^1^ Life Sciences Institute, Guangxi Medical University, Nanning, China; ^2^ Key Laboratory of Longevity and Ageing-Related Disease of Chinese Ministry of Education, Centre for Translational Medicine and School of Preclinical Medicine, Guangxi Medical University, Nanning, China; ^3^ Key Laboratory of High-Incidence-Tumor Prevention and Treatment (Guangxi Medical University), Ministry of Education, Nanning, China

**Keywords:** SLC7A11, ovarian cancer, drug resistance, ceRNA, autophagy, prognosis

## Abstract

Drug resistance is the main cause of chemotherapy failure in ovarian cancer (OC), and identifying potential druggable targets of autophagy is a novel and promising approach to overcoming drug resistance. In this study, 131 genes associated with autophagy were identified from three autophagy-related databases, and of these, 14 were differentially expressed in 90 drug-resistant OC tissues versus 197 sensitive tissues according to the Cancer Genome Atlas ovarian cancer cohort. Among these 14 genes, SLC7A11 was significantly decreased in two paclitaxel-resistant OC cells (HeyA8-R and SKOV3-R) and in 90 drug-resistant tissues compared with their controls. *In vitro* overexpression of SLC7A11 significantly increased the sensitivity of HeyA8-R cells to paclitaxel, inhibited colony formation, induced apoptosis, and arrested cell cycle. Further, low SLC7A11 expression was correlated with poor overall survival (OS), progression-free survival (PFS), and post-progression survival (PPS) in 1815 OC patients. Mechanistically, SLC7A11 strongly regulated cell autophagy as a competing endogenous RNA (ceRNA) based on pan-cancer analyses of 32 tumor types. Specifically, as a ceRNA for autophagy genes STX17, RAB33B, and UVRAG, SLC7A11 was strongly and positively co-expressed with these three genes in 20, 12, and 12 different tumors, respectively, in 379 OC tissues and in 90 drug-resistant OC tissues, and the former two were significantly upregulated in SLC7A11-overexpressed HeyA8-R cells. Further, SLC7A11 induced the protein expression of other autophagy genes, such as LC3, Atg16L1, and Atg7, and the expression of the respective proteins was further increased when the cells were treated with paclitaxel. The results strongly suggest that SLC7A11 regulates autophagy *via* ceRNA interactions with the three abovementioned genes in pan-cancer and in drug-resistant OC. Moreover, low expression of STX17 and UVRAG also significantly predicted low OS, PFS, and PPS. The combination of SLC7A11 with STX17 was more predictive of OS and PFS than either individually, and the combination of SLC7A11 with UVRAG was highly predictive of OS and PPS. The above results indicated that decreased SLC7A11 resulted in drug resistance and effected low rates of survival in OC patients, probably *via* ceRNA interactions with autophagy genes, and thus the gene could serve as a therapeutic target and potential biomarker in OC.

## Introduction

Ovarian cancer (OC) is a common gynecological malignancy of the female reproductive system, with the third highest incidence following cervical and uterine cancer (1, 2). Reduction surgery combined with platinum and paclitaxel-centered chemotherapy is a first-line treatment for OC (3), but the frequent occurrence of drug resistance is the main cause of chemotherapy failure (4). Therefore, it is urgent to find an approach to overcome multidrug resistance to improve the survival and prognosis of patients with OC. Among all the approaches, therapeutic treatment targeting cell autophagy is a promising direction. Increasing studies have revealed that changes in cell autophagy are related to drug resistance and, thus, multiple attempts have been made to identify potential ways to overcome cancer cells resistance (5).

SLC7A11 belongs to the human cystine/glutamate transporter gene family (6), and some studies have revealed that the gene is associated with cancer progression and development (7–11). For example, the gene plays an important role in the tumor progression of breast cancer (12), lung adenocarcinoma (13), gastric cancer (14), and other diseases (15–18). Mechanistically, the gene is involved in the regulation of the cell cycle (19), cell apoptosis (20), ferroptosis (21), and autophagy (22) in different cancers. Among the mechanisms mentioned above, autophagy is the process of metabolism and decomposition of intracellular substances, in which damaged proteins or organelles are enveloped by autophagosomes with a double membrane structure and then enter lysosomes for degradation and recycling (23). Studies have shown that autophagy significantly mediates cancer progression (24), and in ovarian cancer, autophagy has been shown to be associated with tumor metastasis (25), invasion (26), and mediation of drug resistance (27).

Competing endogenous RNAs (ceRNAs) are RNA transcripts that compete with microRNAs (miRNA) at miRNA response elements (MREs) binding sites (28). Thus, RNA molecules that share MREs can regulate each other by competing for miRNA binding (29, 30). ceRNAs include long non-coding RNA (lncRNA), circRNA pseudogenes, and mRNA. While greater emphasis has been placed on non-coding RNAs (31), only a few studies have focused on mRNAs. For example, the tumor suppressor PTEN is regulated by the coding-independent ceRNAs VAPA and CNOT6L (28). Further, our previous study also indicated that NCALD could potentially regulate OC drug resistance by acting as a ceRNA for CX3CL1 (32). However, as described above, the role of SLC7A11 in the modulation of OC is unclear, and its relationship with drug resistance was rare. In this study, based on experimental explorations, big data studies, and comprehensive bioinformatics analyses, we demonstrate the role and mechanism of SLC7A11 in the regulation of OC drug resistance through cell autophagy. This study provides a theoretical basis for the application of SLC7A11 in molecular targeted therapy and in the prediction of treatment outcomes of OC patients.

## Methods

### Cell Lines and Cell Culture

Human OC cell lines were cultured in our laboratory. Cell lines HeyA8 and SKOV3 were kind gifts from Prof. Fengxia Xue of Tianjin Medical University (33). The paclitaxel-resistant OC cells (HeyA8-R and SKOV3-R) were established from parental cell lines (HeyA8 and SKOV3) by gradual exposure to increasing concentrations of paclitaxel. All four OC cell lines were cultured in RPMI-1640 medium (Wisent corporation, Nanjing, China) with 10% fetal bovine serum (FBS) (10099141, Thermo Fisher Scientific, Thornton, NSW, Australia) at 37°C. The resistance index [RI = IC_50_ (paclitaxel-resistant cells)/IC_50_ (parental cells)] of paclitaxel was 5.42 ± 0.55 for HeyA8-R cells and 34.47 ± 4.84 for SKOV3-R cells. The present study was approved by the Ethics Committee of Guangxi Medical University.

### Real-Time Quantitative Polymerase Chain Reaction

TRIzol (Thermo Fisher Scientific, Waltham, Mass, USA) and the NanoDrop 2000 spectrophotometer (Thermo Scientific) were used to isolate and quantify the total RNA. A Primer Script RT reagent kit with gDNA Eraser (RR047A, Takara Biomedical Technology, Beijing, China) was used to synthesize the complementary DNA. The gene expression was measured by the real-time quantitative polymerase chain reaction (RT-qPCR) method using the PowerUp SYBR Green Master Mix (A25742, Thermo Fisher Scientific) and the ABI 7300 System (Thermo Fisher Scientific). The internal reference gene GAPDH was used as a control. The 2^–ΔΔ^ *^CT^* method (34, 35) was used to calculate the expression of the target gene. The primer sequences for SLC7A11 were 5’-TGCCCAGATATGCATCGTCC-3’ and 5’-TCTTCTTCTGGTACAACTTCCAGT-3’. The primer sequences for GAPDH were 5’- CAGCCTCAAGATCATCAGCAAT- 3’ and 5’ -AGTCCTTCCACGATACCAAAGT -3’.

### Western Blotting

According to the results of the CCK-8 and colony formation assays, we found that the sensitivity of SLC7A11 overexpressed cells to paclitaxel was significantly enhanced when the concentration of paclitaxel reached 15.625 nmol/L. Therefore, the cells were treated with 0, 15.625, or 31.25 nmol/L paclitaxel for 72 hours to analyze the effect of paclitaxel on the expression levels of related proteins by western blotting. RIPA protein lysis buffer (Beyotime Inst Biotech, Shanghai, China) and a Pierce*™* BCA Protein Assay Kit (Thermo Fisher Scientific) were used to extract and quantify the proteins. The antibodies were used as follows: SLC7A11 (ab37185, Abcam, Cambridge, UK), p21 Waf1/Cip1 (CST #2947, Cell Signaling Technology, Boston, Mass, USA), p27 Kip1 (CST #3686), CDK2 (CST #2546), CDK7 (CST #2916), Cyclin A2 (CST #4656), Cyclin B1 (CST #12231), Cyclin D3 (CST #2936), LC3 A/B (CST #12741), STX17 (YN4187, Immunoway, Plano, TX, USA), RAB33B (YN1177, Immunoway), UVRAG (CST #13115), Atg 7 (CST#8558), Atg16L1 (CST #8089), Akt (CST #4691), β-Tubulin (CST #2128), GAPDH (CST #5174), and anti-rabbit IgG, and an HRP-linked secondary antibody (CST #7074). Finally, the blots were visualized using the chemiluminescent substrate (ECL) (34580, Thermo Fisher Scientific) and the MiniChemi™ Chemiluminescence imager (Sage Creation, Beijing, China).

### Lentivirus Transfection

The SLC7A11-overexpressed lentiviral particles LV-EF1a > hSLC7A11-CMV > eGFP/T2A/Puro and the control lentiviral particles LV-CMV > eGFP/T2A/Puro were purchased from Cyagen Biosciences (Guangzhou, China). The HeyA8-R cells were seeded into a 96-well culture plate with 2.0 × 10^3^ cells per well at 37°C for 24 hours. Once cells reached a confluence of 30%–50%, they were infected with lentivirus for 24 hours. After 72 hours, RPMI-1640 medium containing 1 µg/mL puromycin was used for selecting cells for 2 weeks. RT-qPCR and western blotting studies were performed to analyze the transfection efficiency of HeyA8-R-eGFP (H-R-eGFP) and HeyA8-R-SLC7A11 (H-R-SLC7A11) cells.

### CCK-8 Assays

The effects of paclitaxel on the cell viability of H-R-eGFP and H-R-SLC7A11 expression were analyzed using the CCK-8 assay (Solarbio, Beijing, China) in four replicate wells. H-R-eGFP and H-R-SLC7A11 cells were both seeded into 96-well culture plates with 600 cells per well at 37°C with 5% CO_2_ in a humidified incubator. After 16 hours of incubation, the cells were treated with varying concentrations of paclitaxel (250, 125, 62.5, 31.25, 15.625, 7.8125, and 3.90625 nmol/L) for 72 hours. The RPMI-1640 medium with treated cells was used as a negative control, and the RPMI-1640 medium without cells was used as a blank control. A total of 10 µL/well of CCK-8 (Solarbio) was added and the 96-well plates were incubated for 2 hours at 37°C. The absorbance (at 450 nm) was calculated by Multiskan GO Microplate Spectrophotometer (Thermo Fisher Scientific). Cell viability (%) = (OD_treated_-OD_blank_)/(OD_control_-OD_blank_) ×100%.

### Colony Formation Assay

H-R-eGFP and H-R-SLC7A11 cells were seeded into 6-well plates with 200 cells per well. After 16 hours of incubation at 37°C, the cells were treated with varying concentrations of paclitaxel (31.25, 15.625, 7.8125, 3.90625, and 1.953125 nmol/L) and RPMI-1640 medium for 8 days, during which the medium was changed every 2 days. The cells were fixed with 4% paraformaldehyde fix solution (Solarbio) for 30 min and dyed with 0.02% crystal violet (Solarbio). The results were scanned by CanonScan 9000F (Canon, Tokyo, Japan), and the colony area was calculated using ImageJ software (National Institutes of Health, Bethesda, MD USA).

### Flow Cytometry

For cell apoptosis analysis, H-R-eGFP and H-R-SLC7A11 cells in the logarithmic growth phase were seeded into 6-cm cell culture dishes. The next day, the cells were treated with varying concentrations of paclitaxel (62.5, 31.25, and 15.625 nmol/L) or RPMI-1640 medium at 37°C for 72 hours. The cells were washed twice with cold PBS (Solarbio) and then resuspend in 1X Binding Buffer at a concentration of 1 x 10^6^ cells/mL. A 5-µL volume of PE Annexin V and 5 µL 7-AAD (Becton, Dickinson and Company, Franklin, NJ, USA) was added. Cells were gently vortexed and then incubated for 15 min at room temperature (25°C) in the dark. Then 400 µL of 1X Binding Buffer was added into each tube. The samples were analyzed by flow cytometry (CytoFLEX, Beckman, California, USA) within 1 hour. Cells that stained positive for PE Annexin V and negative for 7-AAD were undergoing apoptosis. Cells that stained positive for both PE Annexin V and 7-AAD were either in the end-stage of apoptosis and are undergoing necrosis, or were already dead. Cells that stained negative for both PE Annexin V and 7-AAD were viable cells and were not undergoing measurable apoptosis.

For the cell cycle analysis, H-R-eGFP and H-R-SLC7A11 cells were treated with varying concentrations of paclitaxel (62.5, 31.25, 15.625, and 7.8125 nmol/L) or RPMI-1640 medium at 37°C for 72 hours. Harvested cells were washed with pre-cooled Dulbecco’s phosphate-buffered saline (DPBS) and centrifuged to remove supernatant. Then 500 µL DNA staining solution and 5 µL Permeabilization solution (MultiSciences, Hangzhou, China) were added to each sample, and the samples were incubated for 30 min at room temperature (25°C) in the dark. The samples were evaluated by flow cytometry (CytoFLEX, Beckman, CA, USA) and the results were analyzed with ModFit LT software (Verity Software House, Topsham, ME, USA).

### Data Acquisition

The Autophagy Database (http://autophagy.info/) (36) and Thanatos database (http://thanatos.biocuckoo.org/) (37) were used to retrieve autophagy-related genes and proteins. Coremine Medical (http://www.coremine.com/medical/) (38) was utilized for text-mining of autophagy and OC drug resistance-related genes and proteins, using the keywords “autophagy” [‘Autophagy’ (biological process) (40666 connections)], “ovarian cancer” [‘Ovarian Neoplasms’ (mesh) (44253 connections), ‘Malignant neoplasm of ovary’ (disease) (39953 connections)], “drug resistance” [‘Drug Resistance’ (mesh) (54595 connections), ‘Drug Resistance, Multiple’ (mesh) (32082 connections), and ‘Drug Resistance, Neoplasm’ (mesh) (35787 connections)] (*P<*0.05). The database was also used to text-mine drug resistant-related genes and proteins in OC, using the keywords “drug resistance” and “ovarian cancer” (*P<*0.001). The gene expression in 90 drug-resistant OC tissues and 197 drug-sensitive tissues of TCGA ovarian cohort (39) was obtained from cBioPortal (http://www.cbioportal.org) (40, 41). The mRNA expression data and survival information of 1815 OC patients, which included 1656 samples with OS data, 1435 samples with PFS data, and 782 samples with PPS data, were retrieved from Kaplan–Meier Plotter (http://kmplot.com/analysis/index.php?p=service&cancer=ovar) (42), which integrated from 14 microarrays of GEO profiles and TCGA ovarian cancer cohorts.

### Bioinformatics Analyses

The GeneMANIA online tool (http://www.genemania.org/) (43), which collects hundreds of datasets from GEO, BioGRID, Pathway Commons, 12D, and organism-specific functional genomics databases, was used to build protein interaction network. The gene expression data from StarBase (http://starbase.sysu.edu.cn/starbase2/index.php) (44) was used to investigate ceRNA interactions based on gene expression data of 32 types of cancers derived from 10,882 RNA-seq and 10,546 miRNA-seq datasets. The ceRNA pairs were identified as described according to StarBase (44). HITSCLIP, PAR-CLIP, iCLIP, and CLASH data retrieved from the Gene Expression Omnibus were used to determine Ago biding sites; the conserved miRNA target sites were predicted by five miRNA-mRNA interaction tools (TargetScan, RNA22, PITA, Pictar2, and miRanda); then the intersection of Ago biding sites and predicted miRNA target sites were calculated to obtain the CLIP-supported sites, and finally the CLIP-supported miRNA-mRNA interactions were combined (44). The hypergeometric test (45) was used to predict ceRNA pairs among mRNAs, and all ceRNA pairs with FDR<0.05 were included. The ceRNA network was constructed using Cytoscape (National Institutes of Health, Bethesda, MD USA) (46). KEGG (https://www.kegg.jp/) (47) was used to retrieve autophagy genes from the “autophagy-animal” pathway (map04140).

### Statistical Analysis

The data were analyzed using SPSS v23.0 software (IBM, Armonk, NK, USA). All data are represented as the mean values ± SD. The homogeneity of variance was calculated by *t*-test. The bivariate correlations method was used to analyze the correlation between mRNA expressions. The Kaplan–Meier method was utilized for survival analysis, and the auto-selected best cutoff was used to dichotomize gene expression into high and low. Statistically significant difference was indicated by *P<*0.05 (*, *P*<0.05; **, *P*<0.01; ***, *P<*0.001).

## Results

### SLC7A11 Was Decreased in Drug-Resistant Tissues and Predicted Poor Prognosis in OC

#### Fourteen Autophagy-Related Genes Were Deferentially Expressed in Drug-Resistant OC Tissues

**Figure 1:**
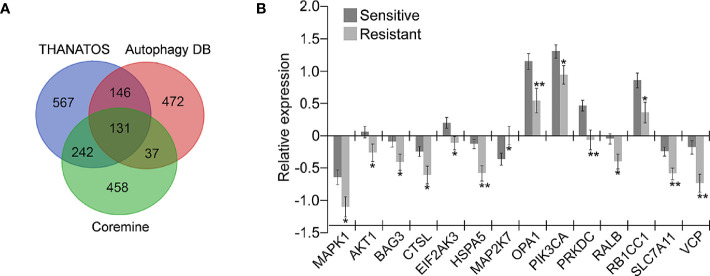
Identification of genes potentially related to autophagy and drug resistance in ovarian cancer (OC). **(A)** The 131 overlapping genes involved in autophagy and drug resistance were retrieved from three independent databases. A total of 786 and 1086 autophagy-related genes were retrieved from database Autophagy DB and Thanatos, respectively. 868 genes related to OC drug resistance and autophagy were selected through text mining conducted by Coremine (P < 0.05); **(B)** Fourteen of the above 131 genes were significantly dysregulated in chemoresistant OC tissues (Resistant, n=90) compared with the sensitive tissues (Sensitive, n=197) according to the TCGA ovarian cohort (*P < 0.05; **P < 0.01).

A total of 786 genes potentially associated with autophagy from the Autophagy Database (36) and 1086 genes from the Thanatos database (37) were acquired; and 868 genes related to autophagy and OC drug resistance were obtained from Coremine Medical (38). The intersection of the above three gene sets was performed, and 131 overlapping genes potentially associated with autophagy and drug resistance were finally obtained (**Figure 1A**). The mRNA level of the 131 genes in 197 chemotherapy-sensitive and 90 resistant OC tissues in the TCGA ovarian cohort was further measured. As shown in **Figure 1B**, 14 of the 131 genes were differentially expressed in the 90 resistant tissues in comparison with the 197 sensitive tissues. Among these, MAP2K7 was significantly upregulated in drug-resistant OC tissues and the remaining genes including AKT1, BAG3, CTSL, EIF2AK3, HSPA5, MAPK1, OPA1, PIK3CA, PRKDC, RALB, RB1CC1, SLC7A11, and VCP were significantly downregulated.

#### Low Levels of Three Genes, SLC7A11, VCP, and OPA1, Predicted Short Survival of OC Patients

**Figure 2:**
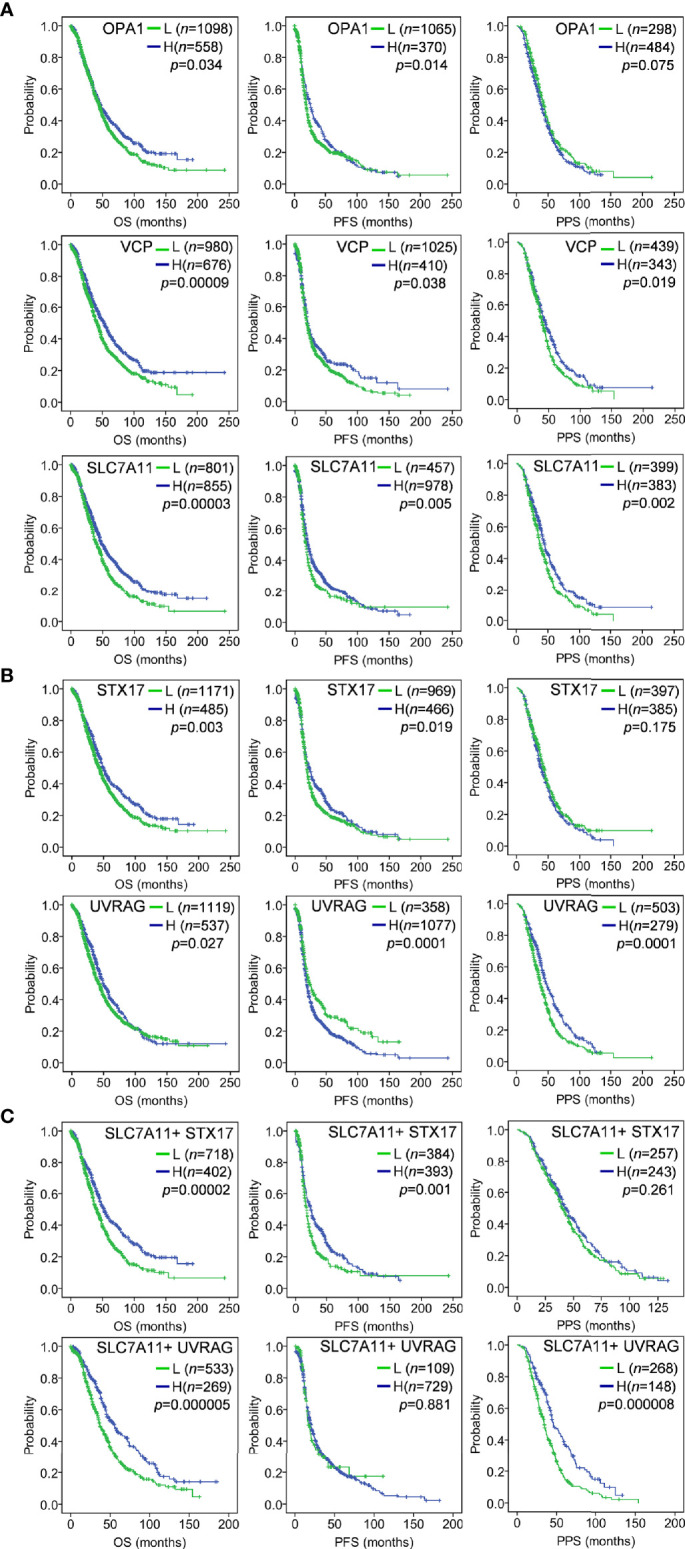
Kaplan–Meier analysis of gene expression with OC survival in a large sample of 1815 OC patients using the Kaplan–Meier Plotter tool. The auto-selected best cutoff value was used to dichotomize gene expression into high (H) and low (L). **(A)** Low expression of OPA1, VCP and SLC7A11 predicted poor OS, PFS and PPS; **(B)** As ceRNA target of SLC7A11, STX17 and UVRAG suppression predicted poor OS, PFS and/or PPS; **(C)** The combination of SLC7A11 expression with STX17 or UVRAG was associated with poor OS, PFS and/or PPS. OS, overall survival; PFS, progression-free survival; PPS, post-progression survival.

The associations of the 14 genes identified in OC were further determined by prognostic analyses in a large sample of 1815 OC patients, which including 1656 samples with OS data, 1435 samples with PFS data, and 782 samples with PPS data, using Kaplan–Meier Plotter (42). Among the 14 genes, several genes such as HSPA5 and PRKDC presented abnormal expression associated with poor OS. Specifically, three genes SLC7A11, VCP, and OPA1 were down-regulated in drug-resistant tissues (**Figure 1B**), and their low expression was significantly associated with poor prognosis. As shown in **Figure 2A**, low expression of SLC7A11 and VCP was significantly related to poor OS, PFS, and PPS, and OPA1 predicted poor OS and short PFS.

### SLC7A11 Contributed to Drug Resistance in OC

#### Up-Regulation of SLC7A11 Enhanced Sensitivity of OC Cells to Paclitaxel

**Figure 3:**
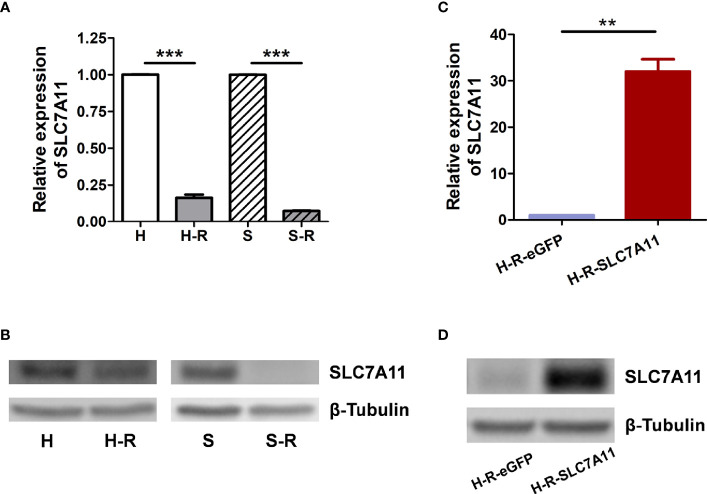
Expression of SLC7A11 in ovarian cancer cell lines. **(A)** RT-qPCR analysis of SLC7A11 mRNA levels in paclitaxel resistant cells HeyA8-R (H-R), SKOV3-R (S-R) and their parental cells HeyA8 (H), SKOV3 (S); **(B)** Western blotting analysis of SLC7A11 expression levels in H-R, H, S-R and S cells; **(C)** Analysis of SLC7A11 mRNA expression in SLC7A11-overexpressed HeyA8-R-SLC7A11 (H-R-SLC7A11) cells and the control HeyA8-R-eGFP (H-R-eGFP) cells by RT-qPCR; **(D)** Western blotting analysis of SLC7A11 expression levels in HeyA8-R-SLC7A11 (H-R-SLC7A11) cells and the control HeyA8-R-eGFP (H-R-eGFP) cells. Values represent the mean ± SD (**P < 0.01; ***P < 0.001).

**Figure 4:**
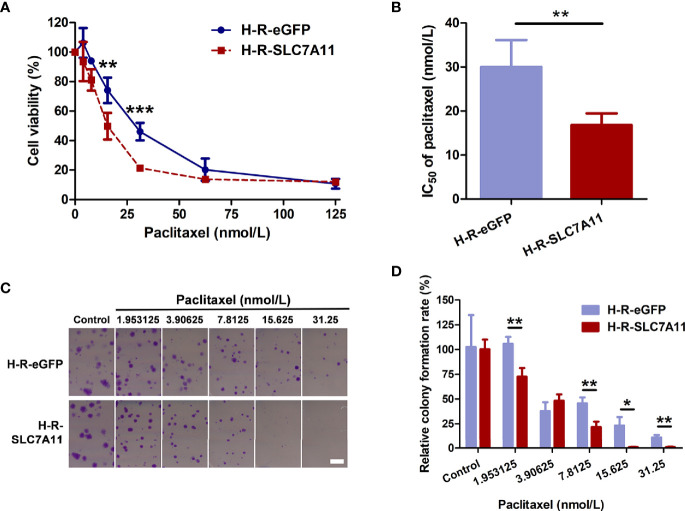
SLC7A11-overexpressed HeyA8-R-SLC7A11 (H-R-SLC7A11) cells were more sensitive to paclitaxel than H-R-eGFP cells. **(A, B)** H-R-SLC7A11 and H-R-eGFP cells were treated with various doses of paclitaxel for 72 hours. The CCK-8 assay was used to reveal the cell viability and the IC_50_ of paclitaxel. Values represented the mean ± SD of four independent experiments (**P < 0.01; ***P < 0.001); **(C)** Representative scan images of the colony formation assay. H-R-SLC7A11 and H-R-eGFP cells were treated with gradient of paclitaxel for 8 days. Scale bar: 4 mm; **(D)** The relative colony formation rates of H-R-SLC7A11 and H-R-eGFP were analyzed by Image J. Values represented the mean ± SD of three independent experiments (*P < 0.05; **P < 0.01).

The mRNA expression of the three genes SLC7A11, VCP, and OPA1 in paclitaxel-resistant OC cells was further measured. As determined by RT-qPCR and western blotting, SLC7A11 mRNA was significantly down-regulated in paclitaxel-resistant HeyA8-R (H-R) and SKOV3-R (S-R) cells in comparison to the HeyA8 (H) and SKOV3 (S) parental cells (**Figures 3A, B**). Thus, further investigations were performed to explore the relationships between SLC7A11 with paclitaxel resistance. The HeyA8-R-SLC7A11 cells were engineered to stably overexpress SLC7A11 (**Figures 3C, D**). The CCK-8 assay showed that SLC7A11-overexpressing HeyA8-R-SLC7A11 cells were more sensitive to paclitaxel compared with the control HeyA8-R-eGFP cells (**Figure 4A**). The IC_50_ value of paclitaxel in H-R-eGFP and H-R-SLC7A11 cells are shown in **Figure 4B**, and the IC_50_ for paclitaxel in H-R-SLC7A11 cells was 1.78 ± 0.22 times less than the control H-R-eGFP cells. Furthermore, the colony formation assay demonstrated that under the same concentration of paclitaxel treatments, the relative colony formation rate of SLC7A11 overexpressed HeyA8-R-SLC7A11 cells was significantly lower than that of HeyA8-R-eGFP cells (**Figures 4C, D**). These results suggested that SLC7A11 overexpression increased the paclitaxel sensitivity of paclitaxel-resistant OC cells.

#### Up-Regulation SLC7A11 Further Induced Cell Apoptosis of H-R Cells After Paclitaxel Treatments

**Figure 5:**
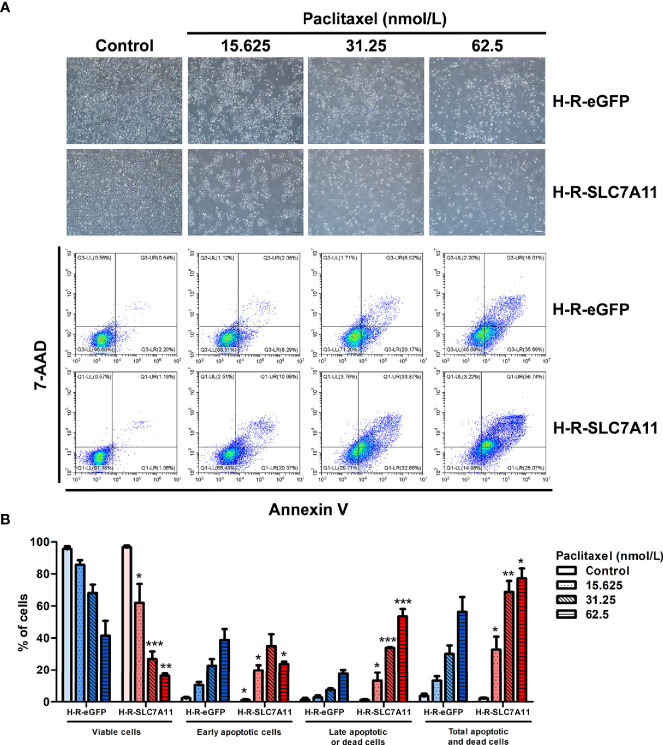
Effects of paclitaxel on the apoptosis of H-R-eGFP and H-R-SLC7A11 cells by PE Annexin V/7-AAD-double staining flow cytometry analysis. **(A)** Representative cell-culture images and flow cytometric findings of H-R-eGFP and H-R-SLC7A11 cells treated with gradient concentrations of paclitaxel for 72 hours. The lower left quadrant shows living cells, the lower right quadrant early apoptotic cells, the upper left quadrant non-apoptotic dead cells, and the upper right quadrant shows late apoptotic cells and dead cells; **(B)** The histogram of apoptosis experiments. Statistical analysis was performed to compare the living cell rate of H-R-eGFP to H-R-SLC7A11 (lower left quadrant), early apoptotic cell rate (lower right quadrant), late apoptotic cell rate, and dead cell rate (upper right quadrant), and total apoptosis and death cell rates (upper left + upper right + lower right quadrant). The statistical analysis in the figure was a pair-wise comparison between H-R-eGFP and H-R-SLC7A11 cells treated with the same paclitaxel concentration and the same state of cells (*P < 0.05, **P < 0.01, ***P < 0.001).

Cell apoptosis was analyzed by flow cytometry following H-R-SLC7A11 and H-R-eGFP treatment with paclitaxel for 72 hours. With the increasing paclitaxel concentration, the percentage of living cells in H-R-SLC7A11 cells decreased more significantly compared to that of H-R-eGFP cells. Following paclitaxel treatment, the percentage of early apoptotic cells in H-R-SLC7A11 was higher than that in H-R-eGFP cells, and the percentage of total apoptotic and dead cells in H-R-SLC7A11 was also significantly higher than that in H-R-eGFP cells (**Figure 5**). Our results indicated that SLC7A11 overexpression could further increase the apoptosis and mortality rate of OC cells by paclitaxel treatments.

#### Up-Regulation of SLC7A11 Further Blocked the Cell Cycle of H-R Cells After the Addition of Paclitaxel

**Figure 6:**
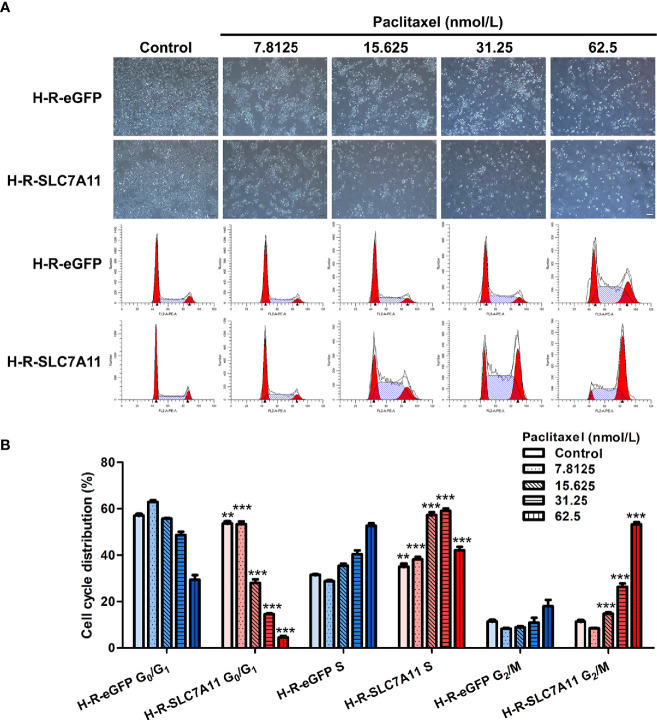
Effects of paclitaxel on the cell cycle distribution of H-R-eGFP and H-R-SLC7A11 by PI staining and the flow cytometry analysis. **(A)** Representative cell-culture photos and cell-cycle images for H-R-eGFP and H-R-SLC7A11 cells treated with gradient paclitaxel for 72 h; **(B)** The percentage of cells in G0/G1 phase, S phase and G2/M phase of H-R-eGFP and H-R-SLC7A11 cells treated with gradient concentrations of paclitaxel for 72 hours. The statistical analysis in the figure was a pair-wise comparison between H-R-eGFP and H-R-SLC7A11 cells data with the same paclitaxel concentration and the same cell-cycle stage (**P < 0.01, ***P < 0.001).

**Figure 7:**
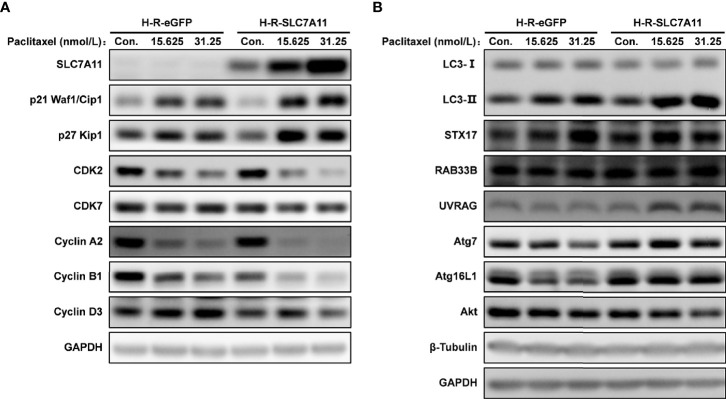
Effects of SLC7A11 and paclitaxel on the expression of related proteins analyzed by western blotting. The H-R-eGFP and H-R-SLC7A11 cells were treated with gradient concentration of paclitaxel for 72 hours. **(A)** Proteins were extracted and detected by western blotting to analyze the expression of p21 Waf1/Cip1, p27 Kip1, CDK2, CDK7, Cyclin A2, Cyclin B1 and Cyclin D3; **(B)** Expression of LC3 -II, LC3 -I, STX17, RAB33B, UVRAG, Atg7, Atg16L1 and Akt proteins were analyzed by western blotting. Representative blots were shown with GAPDH and β-Tubulin as loading control.

The cell cycle was analyzed by flow cytometry following the treatment of H-R-SLC7A11 and H-R-eGFP with paclitaxel for 72 hours. With the increasing concentrations of paclitaxel (0, 7.8125, 15.625, 31.25, and 62.5 nmol/L), the proportion of G_0_/G_1_ phase cells in H-R-SLC7A11 was significantly decreased compared with H-R-eGFP, while the proportion of cells in the S phase was significantly increased (**Figure 6**). The cell cycle arrest of H-R-SLC7A11 was more obvious than that of H-R-eGFP, indicating that the cell cycle of H-R-SLC7A11 with overexpression of SLC7A11 was more easily blocked by paclitaxel than that of H-R-eGFP. These findings suggested that SLC7A11 overexpression could reduce paclitaxel resistance of ovarian cancer drug-resistant cells H-R by regulating the cell cycle. Due to the above changes in the cell cycle, the expression of cell cycle proteins in H-R-eGFP and H-R-SLC7A11 cells were analyzed by western blotting. As shown in **Figure 7A**, after 72 hours of paclitaxel treatment, the expression of the cell-cycle inhibitors p21 Waf1/Cip1 and p27 Kip1 were significantly higher compared to control cells under various concentrations of paclitaxel. Furthermore, paclitaxel significantly inhibited the expression of the cell cycle promoting proteins CDK2, CDK7, Cyclin A2, Cyclin B1, and Cyclin D3 in H-R-SLC7A11 cells compared with H-R-eGFP cells. The results showed that the cell cycle of H-R-SLC7A11 cells was more easily blocked by paclitaxel than H-R-eGFP cells at cellular and protein levels. These results were consistent with the results of cell viability assay, colony formation, and cell apoptosis analyses, showing that the up regulation of SLC7A11 enhanced the sensitivity of OC cells to paclitaxel.

#### SLC7A11 Significantly Interacted With Drug Resistance Regulatory Proteins in OC

**Figure 8:**
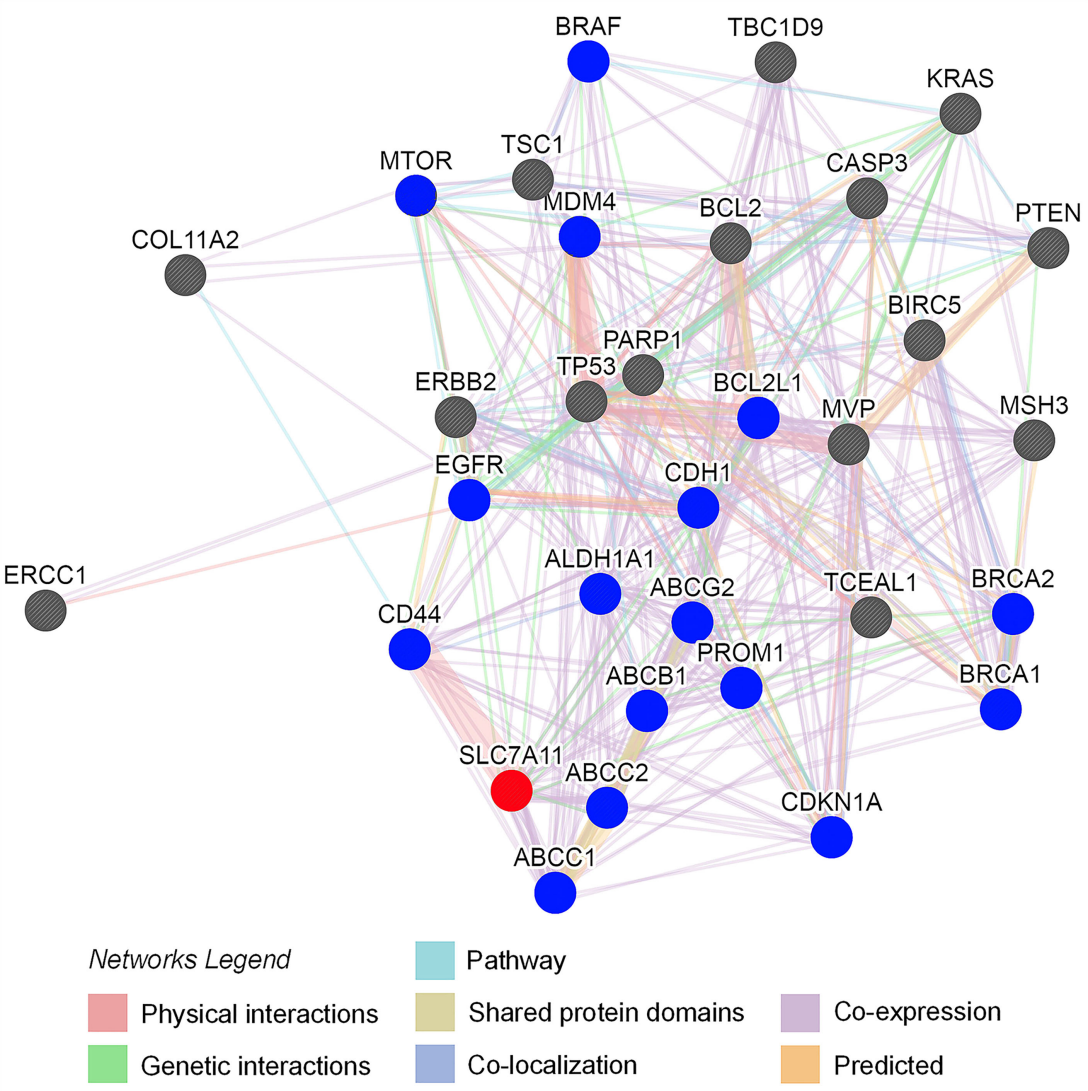
Protein interaction network of SLC7A11 created by the GeneMANIA online tool. The 31 drug resistance-related proteins were text-mined by Coremine Medical (*P < *0.001). The blue circles indicate the 16 proteins which interacted with SLC7A11 directly; the grey circles indicate the proteins which interacted with SLC7A11 indirectly. The interaction types between proteins indicated as networks legend.

Protein-protein interaction (PPI) analysis was conducted to evaluate the association between SLC7A11 expression with drug resistance. Thirty-one proteins contributing to drug resistance in OC were retrieved using the Coremine Medical tool (*P<*0.001), and the PPI network of SLC7A11 with the 31 proteins was generated. As shown in **Figure 8**, the PPI network indicated that SLC7A11 directly interacted with 16 proteins, and indirectly interacted with the remaining 15 proteins. All the 16 proteins have been reported to be implicated in the modulation of drug resistance in OC, namely ABCC1 (48), ABCC2 (49), ABCB1 (50), CD44 (51), ALDH1A1 (52), ABCG2 (53), PROM1 (54), CDKN1A (55), CDH1 (56), EGFR (57), BRCA1 (58), BRCA2 (58), BCL2L1 (59), MDM4 (60), MTOR (61), and BRAF (62); and among the 15 proteins, nine of them were reported to be implicated in drug resistance, which were ERCC1 (63), MVP (64), ERBB2 (65) TP53 (66), PARP1 (58), MSH3 (67), BCL2 (68), CASP3 (69), and PTEN (70). The results above indicated that among the 31 proteins, SLC7A11 interacted with 25 OC drug resistance-related proteins, providing strong PPI network evidence to support the relationship between SLC7A11 and drug resistance in OC.

### SLC7A11 Positively Regulated Autophagy in OC Drug Resistance as a ceRNA

#### SLC7A11 to Exert Its Biological Roles in Cancer *via* ceRNA

The above results suggested that SLC7A11 was potentially an autophagy-related gene that was implicated in the regulation of drug resistance and affected prognosis in OC. Thus, further analyses were performed to define the relationship between SLC7A11 with autophagy in OC progression. Based on the pan-cancer analyses using the StarBase tool (44) that includes 32 tumors types, the ceRNA analyses of SLC7A11 with 109 genes in the “autophagy-animal” pathway (map04140) was systematically analyzed. ceRNA networks from large-scale interactions of miRNA-targets which were identified based on CLIP-seq data of pan-cancer analysis were performed. The results indicated that SLC7A11 could potentially regulate 4595 genes acting as a ceRNA in 32 types of cancers.

#### SLC7A11 Strongly Regulated Autophagy in OC and in 31 Other Cancer Types as a ceRNA

**Figure 9:**
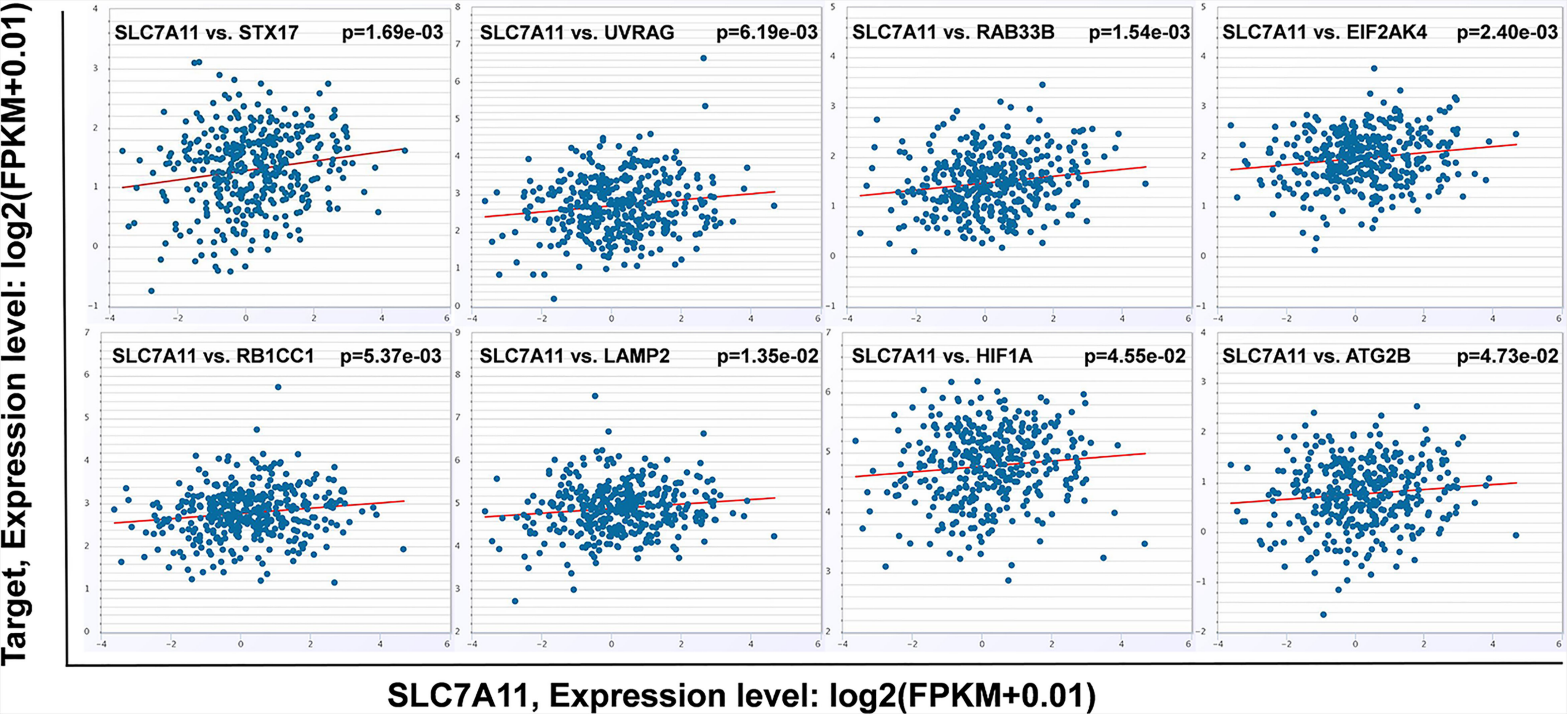
The relationship between SLC7A11 and eight autophagy related genes. SLC7A11 act as a ceRNA for eight autophagy genes and positively co-expressed with these genes in 379 ovarian cancer tissues of TCGA cohort according to StarBase (*P<*0.05). The gene expression values from RNA-seq data were presented as log2 (FPKM + 0.01).

**Figure 10:**
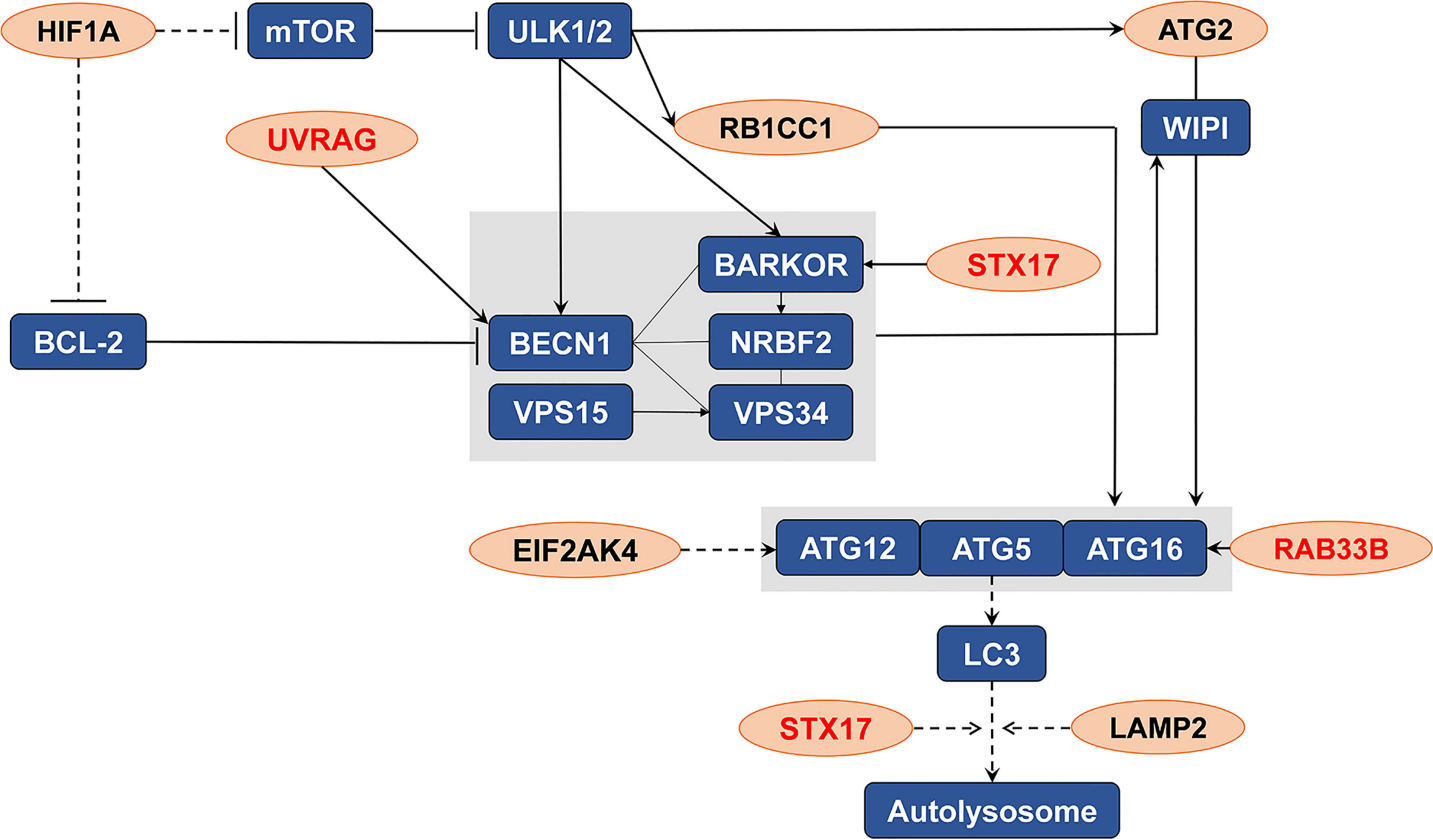
The distribution of the eight genes in autophagy pathway (map04140). The eight genes (indicated as orange oval) were the potential targets of SLC7A11 as a ceRNA in 379 ovarian cancers which significantly and positive co-expressed with the SLC7A11, and the three genes (indicated in red) were those co-expressed in 90 drug resistant ovarian cancer tissues.

An intersection of the 4595 genes and the 109 genes active in the autophagy pathway (map04140) was performed, and 42 overlapping genes were obtained, which accounted for 38.5% of the genes in the autophagy pathway. As shown in **Table 1**, SLC7A11 strongly interacted with 42 autophagy genes as a ceRNA by sponging miRNAs in 32 tumor types. Specifically, SLC7A11 functioned as a ceRNA for eight autophagy genes in OC, which included STX17, UVRAG, RAB33B, EIF2AK4, RB1CC1, LAMP2, HIF1A, and ATG2B (**Table 1**). In line with the proposed mechanism of ceRNA, SLC7A11 was significantly and positively co-expressed with the eight autophagy genes in OC (**Figure 9**), and thus SLC7A11 would positively regulate autophagy (**Figure 10**).

**Table 1:** SLC7A11 regulate 42 autophagy genes functioned as a ceRNA in 32 types tumors.

ceRNA	No. of microRNA*	*P* value	FDR	No. of tumors #	Ovarian cancer
RAB33B	85	7.14E-08	2.63E-07	14	√
STX17	71	1.33E-05	2.24E-05	20	√
HIF1A	72	1.91E-08	8.50E-08	27	√
ATG2B	109	1.57E-07	5.02E-07	15	√
UVRAG	77	5.09E-06	9.77E-06	12	√
EIF2AK4	69	8.30E-08	2.95E-07	18	√
RB1CC1	76	5.41E-08	2.08E-07	21	√
LAMP2	114	1.64E-08	7.45E-08	23	√
PIK3R3	133	9.34E-08	3.25E-07	16	
ITPR1	82	2.00E-08	8.84E-08	17	
KRAS	135	1.38E-07	4.53E-07	26	
NRAS	109	1.73E-08	7.83E-08	24	
VMP1	84	7.54E-09	3.90E-08	23	
EIF2S1	108	3.26E-06	6.63E-06	23	
EIF2AK3	54	2.62E-04	3.10E-04	23	
PDPK1	135	8.58E-07	2.14E-06	20	
SH3GLB1	121	4.13E-14	2.00E-12	20	
MTOR	49	8.11E-04	8.54E-04	20	
PIK3CA	78	1.32E-07	4.40E-07	19	
ATG5	97	1.28E-09	8.87E-09	17	
PIK3R1	157	1.32E-16	2.32E-14	17	
MAP3K7	92	2.45E-09	1.51E-08	17	
ATG12	90	1.81E-06	4.03E-06	17	
IRS1	78	6.96E-06	1.29E-05	17	
WIPI2	81	1.03E-04	1.35E-04	17	
PIK3R4	30	1.86E-04	2.28E-04	17	
BCL2	149	3.71E-08	1.51E-07	15	
MTMR3	120	7.81E-08	2.84E-07	15	
PIK3CB	65	6.70E-05	9.25E-05	15	
RRAS2	67	7.10E-06	1.31E-05	15	
DDIT4	63	3.55E-05	5.30E-05	14	
MTMR4	112	2.28E-06	4.87E-06	13	
RUBCN	119	6.96E-05	9.60E-05	13	
TSC1	143	9.82E-07	2.40E-06	13	
PTEN	145	5.52E-11	6.73E-10	12	
IGF1R	184	1.85E-08	8.24E-08	12	
IRS2	105	5.71E-06	1.08E-05	12	
TP53INP2	106	1.05E-04	1.38E-04	12	
ULK2	67	3.18E-06	6.48E-06	12	
RRAGD	62	1.04E-04	1.37E-04	11	
RAB7A	78	8.87E-10	6.50E-09	9	
SNAP29	93	2.20E-08	9.57E-08	8	

*Number of microRNAs that SLC7A11 interacted with the autophagy gene as a ceRNA.

^#^Number of tumor types that SLC7A11 potentially regulate the autophagy gene as a ceRNA.

#### SLC7A11 Acted as ceRNA for Three Autophagy Genes STX17, UVRAG, and RAB33B, and Induced Autophagy in Drug-Resistant OC Cells

**Figure 11:**
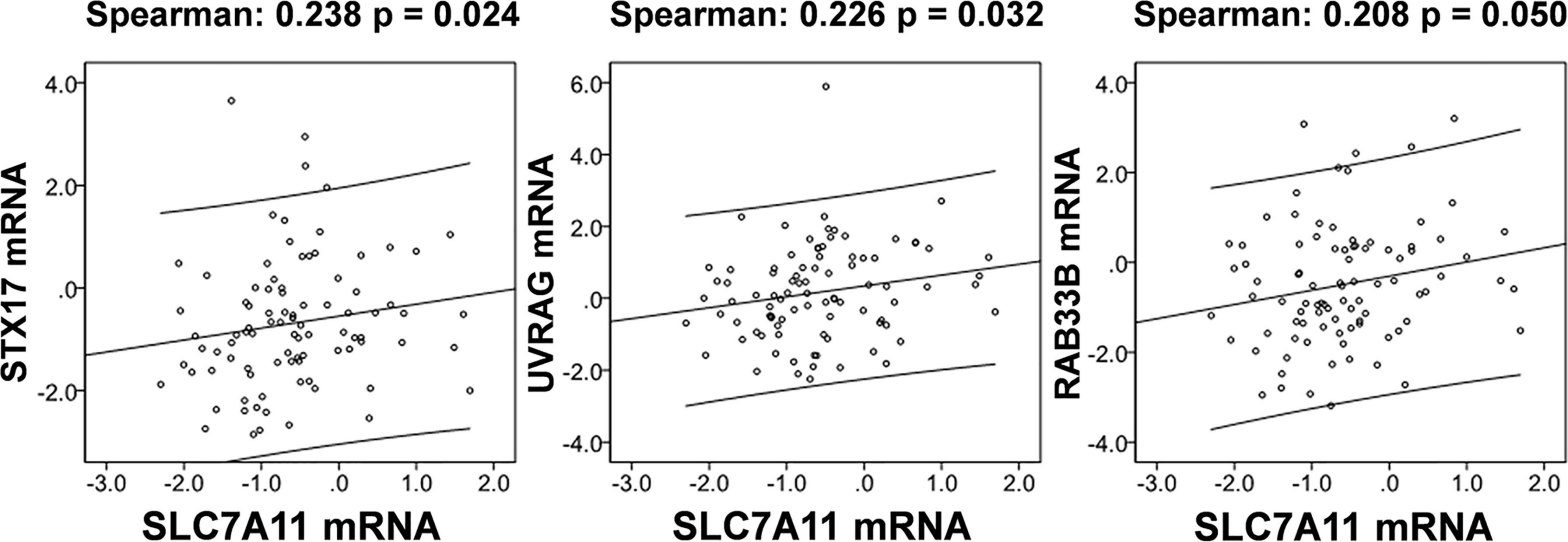
SLC7A11 is positively correlated with the expression of STX17, UVRAG, and RAB33B in 90 drug resistant ovarian cancer tissues based on TCGA cohort. The correlation of genes was analyzed by bivariate correlation (*P < *0.05).

**Figure 12:**
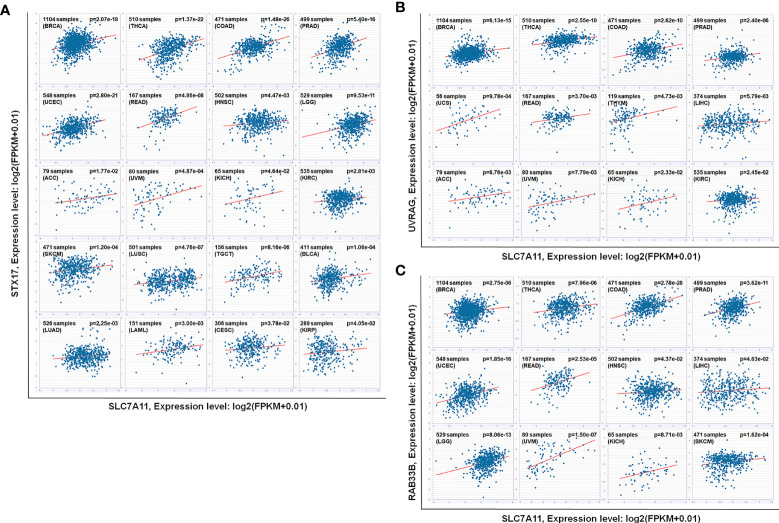
As ceRNA targets of SLC7A11, **(A)** STX17, **(B)** UVRAG, and **(C)** RAB33B were positively co-expressed in 12, 20 and 12 kinds of cancers according to pan-cancer analyses. The other 2 cancers that SLC7A11 negatively co-expressed with RAB33B was excluded. The gene expression values from RNA-seq data were presented as log2 (FPKM + 0.01) (*P<0.05*). BRCA, Breast Invasive Carcinoma; THCA, Thyroid Carcinoma; COAD, Colon Adenocarcinoma; PRAD, Prostate Adenocarcinoma; UCEC, Uterine Corpus Endometrial Carcinoma; READ, Rectum Adenocarcinoma; HNSC, Head and Neck Squamous Cell Carcinoma; LGG, Brain Lower Grade Glioma; ACC, Adrenocortical Carcinoma; UVM, Uveal Melanoma; KICH, Kidney Chromophobe; KIRC, Kidney Renal Clear Cell Carcinoma; SKCM, Skin Cutaneous Melanoma; LUSC, Lung Squamous Cell Carcinoma; TGCT, Testicular Germ Cell Tumors; BLCA, Bladder Urothelial Carcinoma; LUAD, Lung Adenocarcinoma; LAML, Acute Myeloid Leukemia; CESC, Cervical Squamous Cell Carcinoma and Endocervical Adenocarcinoma; KIRP, Kidney Renal Papillary Cell Carcinoma; UCS, Uterine Carcinosarcoma; THYM, Thymoma; LIHC, Liver Hepatocellular Carcinoma.

**Figure 13:**
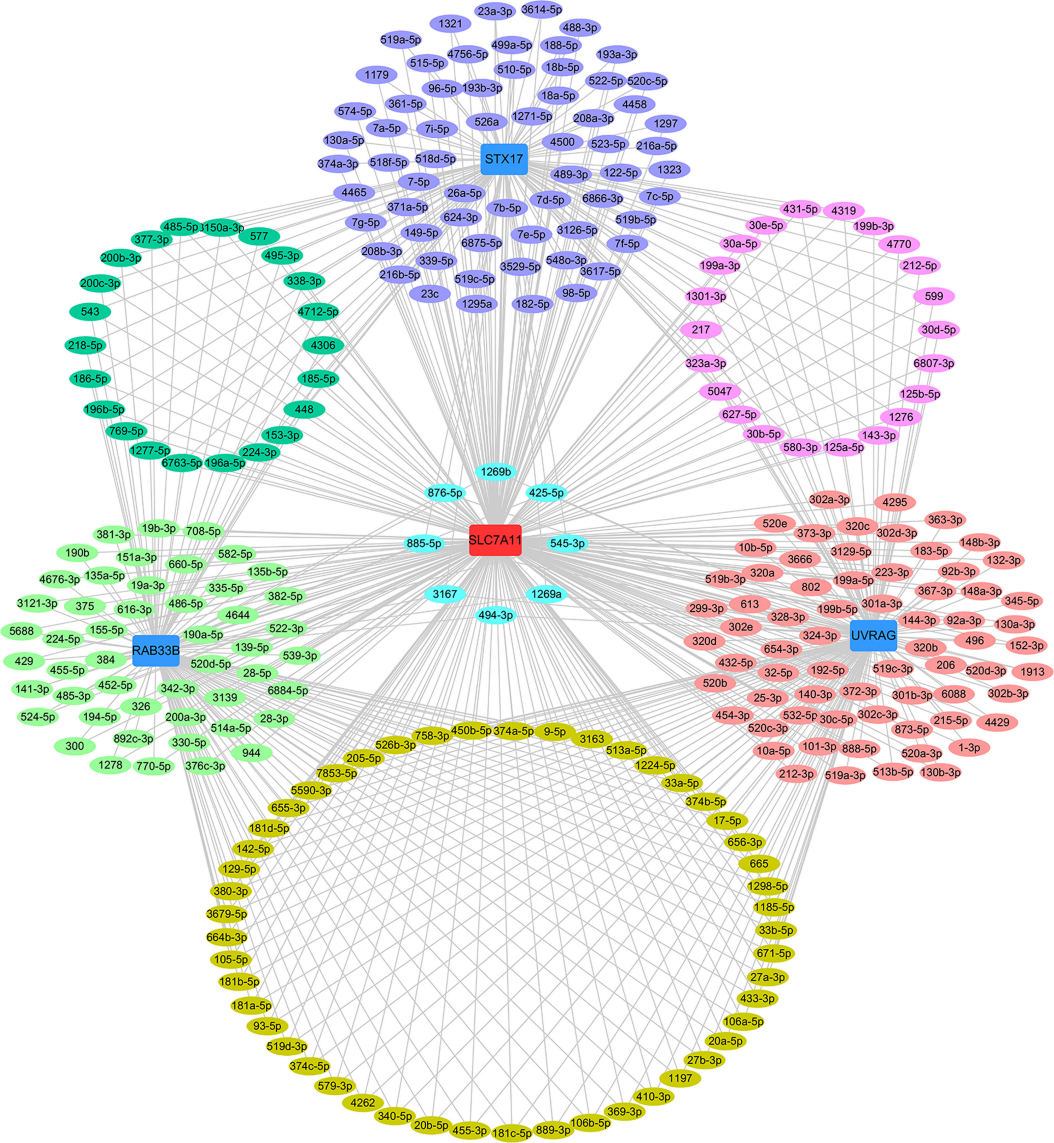
ceRNA network of SLC7A11 with STX17, UVRAG and RAB33B. The ceRNA pairs were determined by StarBase and the network was constructed by Cytoscape. Acting as a ceRNA, SLC7A11 shared miRNAs with the target genes as indicated in the network. The light green oval indicate the miRNAs for SLC7A11 with RAB33B, the purple oval indicates the miRNAs for SLC7A11 with STX17, the rose-colored oval indicates the miRNAs for SLC7A11 with UVRAG, the green oval indicate the common miRNAs for SLC7A11 with RAB33B and STX17, the mauve oval indicates the common miRNAs for SLC7A11 with STX17 and UVRAG, the deep yellow oval indicates the common miRNAs for SLC7A11 with UVRAG and RAB33B, the sky-blue oval indicates the common miRNAs for SLC7A11 with STX17, UVRAG and RAB33B.

The relationships between SLC7A11 and the 8 genes in 90 drug-resistant tissues were evaluated, and we found that SLC7A11 was consistently and positively co-expressed with STX17, UVRAG, and RAB33B in resistant tissues (**Figure 11**). Taken together, SLC7A11 acted as a ceRNA for STX17, UVRAG, and RAB33B in 20, 12, and 12 different tumor types, respectively (**Table 1** and **Figure 12**), and acted as a ceRNA for the three genes in 379 OC tissues (**Table 1** and **Figure 9**) and in 90 drug-resistant tissues (**Figure 11**). Thus, a ceRNA network for SLC7A11 with the three target genes (STX17, RAB33B, and UVRAG) was constructed, and detailed information about the MREs of SLC7A11 with the three genes was obtained. As shown in **Figure 13**, SLC7A11 shared MREs with STX17 covering a total of 117 miRNAs from 71 miRNA families, with UVRAG covering a total of 147 miRNAs from 77 miRNA families, and with RAB33B covering a total of 127 miRNAs from 85 miRNA families. Of these, SLC7A11 shared common MREs with STX17 and UVRAG covering a total of 22 common miRNAs, with UVRAG and RAB33B covering a total of 49 common miRNAs, and with STX17 and RAB33B covering a total of 22 common miRNAs. Most importantly, SLC7A11 shared MREs with all three genes which covered a total of eight common miRNAs.

The role of SLC7A11 as a ceRNA for STX17, UVRAG, and RAB33B in OC drug resistance was further confirmed in H-R-eGFP and H-R-SLC7A11 cells, and the effect of SLC7A11 and paclitaxel on cell autophagy proteins was investigated (**Figure 7B**). Compared with the expression in control H-R-eGFP cells, the expression of STX17 and RAB33B was significantly increased as a result of SLC7A11 overexpression in H-R-SLC7A11 cells, which was consistent with the big data results that SLC7A11 acted as a ceRNA for these genes and was positively co-expressed in up to 20 different tumors, 379 OC tissues, and 90 drug-resistant tissues (**Figures 9**, **11**, **12**). In addition, we revealed that the overexpression of SLC7A11 increased the ratio of LC3-II/I expression. When we treated cells with paclitaxel, we found that the expression levels of SLC7A11 were increased in H-R-SLC7A11 cells but not in H-R-eGFP cells, and autophagy-related proteins, such as LC3-II/I, Atg7, Atg16L1, RAB33B, UVRAG, all were up-regulated in H-R-SLC7A11 cells compared with non-SLC7A11 overexpressing H-R-eGFP cells. In addition, the autophagy-related upstream protein Akt was decreased in H-R-SLC7A11 cells compared to H-R-eGFP cells, and further decreased after paclitaxel treatments.

#### The Combination of ceRNA SLC7A11 With the Target STX17, UVRAG, and RAB33B Showed a Better Prognostic Outcome

Given the ceRNA relationship of SLC7A11 with the three genes (STX17, UVRAG, RAB33B) and the predictive role of SLC7A11 on OC survival, we further analyzed the associations of the three genes with prognosis. As expected, a low expression of STX17 and UVRAG significantly predicted shorter OS, PFS, and PPS in 1815 patients with OC (**Figure 2B**), since SLC7A11 positively co-expressed with these genes, and a low SLC7A11 expression also predicted shorter OS, PFS, and PPS (**Figure 2A**). Moreover, the combination of SLC7A11 with STX17 was more predictive of OS and PFS than any of the genes used individually, and the combination of SLC7A11 with UVRAG was highly predictive of OS and PPS (**Figure 2C**). These results also supported the conclusion that SLC7A11 acts as ceRNA for STX17 and UVRAG.

## Discussion

Drug resistance and postoperative recurrence are the main causes of chemotherapy failure in OC (4), and relieving multidrug resistance can improve the survival and prognosis of patients. Therefore, the identification of potential targets able to overcome drug resistance is the key to reducing patient mortality. SLC7A11 has been reported to be involved in tumor progression, but its correlation with drug resistance in OC was poorly understood. In this study, we verified a role for SLC7A11 in modulation drug resistance and in predicting survival in OC, *via* regulation of cell autophagy as a ceRNA.

SLC7A11 belongs to the glutamate/cystine antiporter solute carrier family, also known as xCT, and plays a variety of roles in the regulation of tumor growth (15), invasion (71), metastasis (71), and unfavorable prognosis (72). For example, SLC7A11 is associated with carcinogenesis, cancer stem cells (CSCs), and poor prognosis in breast cancer (12, 72, 73), and plays a role in recurrence, lymph node metastasis, and venous invasion in colorectal cancer (74). However, the association of SLC7A11 with cancer drug resistance remains poor. Studies have reported that elastin, a ferroptosis inducer, could irreversibly inhibit SLC7A11 and synergize with cisplatin to increase the drug’s cytotoxicity (75), and that the blocking of SLC7A11 effectively increased the intracellular level and cytotoxicity of cisplatin in colorectal cancer (76). In the present study, we revealed that SLC7A11 was significantly decreased in two paclitaxel-resistant OC cell lines and in 90 drug-resistant tissue samples when compared with their respective controls (**Figures 1B**, and **3A, B**). The overexpression of SLC7A11 increased the paclitaxel sensitivity of paclitaxel-resistant ovarian cancer cells HeyA8-R, induced cell apoptosis, and arrested the cell cycle (**Figures 4**–**7A**). Furthermore, shorter OS, PFS, and PPS in a large sample of 1815 patients was predicted by low SLC7A11 expression (**Figure 2A**). These results suggested that a low expression of the gene could lead to shorter survival of OC patients *via* its influence on drug resistance. The SLC7A11 expression on OC drug resistance was further supported by the protein interaction network. The results of PPI analysis indicated that SLC7A11 might play a role in drug resistance by interacting with groups of drug resistance-related proteins, as an interaction of SLC7A11 with 25 OC drug resistance-related proteins was detected (**Figure 8**).

The potential mechanisms involving SLC7A11 in drug resistance in OC were investigated, and a potential role of the gene as a ceRNA on cell autophagy genes was revealed. A pan-cancer analysis of 32 cancer types revealed that SLC7A11 could potentially regulate 4595 genes as a ceRNA, providing strong support for SLC7A11 activity as a ceRNA in tumor regulation. Further, three independent databases determined that SLC7A11 expression was closely related to autophagy (**Figure 1A**), and SLC7A11 was predicted to act as ceRNA for 42 autophagy genes in 32 cancer types (**Table 1**), which account for 38.5% (42/109) of the genes in the autophagy pathway (map04140). The above results provide strong evidence that SLC7A11 regulates cell autophagy in cancer by acting as a ceRNA for autophagy genes. In particular, SLC7A11 was associated as a ceRNA for 8 autophagy genes and was positively co-expressed with those genes in OC (**Table 1** and **Figure 9**). Consistently, SLC7A11 acted as ceRNA for 3 (STX17, UVRAG, and RAB33B) of the 8 genes, and positively co-expressed with these 3 genes in 90 drug-resistant tissues (**Figure 11**). The ceRNA relationship of SLC7A11 with the 3 genes was further confirmed by ceRNA network analysis (**Figure 13**), and was experimentally supported by the measurement of protein expression in H-R-eGFP and H-R-SLC7A11 cells. The results indicated that the expression level of STX17 and RAB33B was significantly increased when SLC7A11 was overexpressed (**Figure 7B**). All these results primarily supported the conclusion that SLC7A11 acted as a ceRNA for autophagy genes, specifically for STX17, UVRAG, and RAB33B in drug-resistant OC.

The positive relationship between SLC7A11 and autophagy was further investigated, and we revealed that the ratio of LC3-II/I expression was increased in H-R-SLC7A11 overexpressing cells, and further increased after paclitaxel treatment (**Figure 7B**). Similarly, other autophagy proteins such as Atg7, Atg16L1, RAB33B, and UVRAG were also upregulated in H-R-SLC7A11 cells following paclitaxel treatment. Collectively, we concluded that as a ceRNA for the autophagy genes STX17, UVRAG, and RAB33B, SLC7A11 might decrease drug resistance in OC by promoting autophagy. The role of SLC7A11 on autophagy has been previously reported, whereby dysregulated SLC7A11 expression inhibited cell growth through the ROS/autophagy pathway in liver cancer (77), but there is no prior evidence of the gene acting as a ceRNA.

Biomarkers are crucial for the prediction of progression, risk stratification, and overall therapeutic benefits, and the accurate survival prediction for OC patients can help guide treatment decisions, and may greatly improve the OS rate and recurrence/recurrence-free survival rate of OC patients (78, 79). Hence, the identification of new genes able to predict prognosis is very important for the treatment of OC. Drug resistance is a major obstacle in the treatment of OC patients and is often linked to relapse and short survival (80). Thus, genes contributing to drug resistance are often associated with poor prognosis. We previously reported that low expression of PDIA4 was implicated in drug resistance and could predict short OS and DFS in OC (81). In this study, we found that autophagy-related genes SLC7A11, OPA1, and VCP were down-regulated in 90 drug-resistant OC tissues versus 197 sensitive tissues (**Figure 1B**), and down-regulation of the 3 genes predicted short OS, PFS, and PPS in large samples including 1815 OC patients (**Figure 2A**). Interestingly, SLC7A11 may represent a potential treatment target as a ceRNA. As potential targets of ceRNA SLC7A11, the low expression of STX17 and UVRAG significantly predicted short OS, PFS, and PPS, and the combination of SLC7A11 with STX17 and UVRAG had better predictive potential for OS than each gene individually (**Figures 2B, C**). Given the large sample size in this study, the above-mentioned 5 genes could be potentially used as prognostic markers in the treatment of OC, particularly the combination of SLC7A11 with STX17 and UVRAG. Indeed, we have been previously reported that the low expression of SLC7A11 predicted short OS as well as DFS in a smaller cohort of 489 OC patients (81); and low levels of VCP in HPV-negative patients was related to worse 5-year DFS in oropharyngeal squamous cell carcinoma (82). These results are essentially consistent with those of the present study and support our findings.

Autophagy is a conserved cellular self-digestive mechanism that is crucial for development and to alleviate multiple environmental stresses (5), and substantial research has suggested that autophagy is involved in the modulation of cancer drug resistance (83). Interestingly, autophagy is a double-edged sword in the regulation of cancer drug resistance. In some contexts, it exerts a cytoprotective effect that leads to treatment resistance, while in others, it has a cytotoxic effect that induces cell death, while the inhibition of autophagy confers drug resistance (84). Thus, novel therapeutic approaches that target autophagy are a promising direction for cancer treatment that may help to overcome drug resistance (85). In OC and other cancers such as lung carcinomas and osteosarcoma, defects in autophagy often play a role in drug resistance to cisplatin, paclitaxel, gemcitabine, and doxorubicin (85). In this study, decreased expression of SLC7A11 in drug-resistant OC tissues likely blocked autophagy *via* inhibition of STX17, UVRAG, and RAB33B expression, which finally resulted in drug resistance and shorter survival (**Figures 2**, **4**, **10**, **11**). Furthermore, 13 other genes—MAP2K7, AKT1, BAG3, CTSL, EIF2AK3, HSPA5, MAPK1, OPA1, PIK3CA, PRKDC, RALB, RB1CC1, and VCP—were dysregulated in 90 drug-resistant OC tissues when compared to 197 sensitive tissues, and were significantly related to autophagy as determined by 3 independent autophagy-related databases, suggesting that those genes may be potentially involved in modulating drug resistance *via* autophagy (**Figure 1**). In fact, some of the above 14 genes have been shown to be involved in the modulation of drug resistance in OC. For instance, decreased BAG3 expression enhances the cisplatin sensitivity of OC cells *via* the inhibition of autophagy (86), and increased AKT1 expression suppresses autophagy (87). The results above suggest that the 14 genes identified in this study may represent potential targets for the treatment of drug-resistant OC through autophagy-dependent techniques, although further studies should be performed to clearly explain the mechanisms involved.

In conclusion, SLC7A11 acts as a ceRNA to regulate the development of tumors by mediating cell autophagy. The overexpression of SLC7A11 improved the sensitivity of OC cells to chemotherapeutic agents, and conversely, the downregulation of SLC7A11 led to drug resistance, probably by inhibiting the expression of autophagy genes STX17, UVRAG, and RAB33B through a ceRNA mechanism which inhibited autophagy and resulted in shorter survival of patients with OC. Moreover, the low expression of STX17, UVRAG, OPA1, and VCP significantly predicts shorter survival outcomes, while the combination of SLC7A11 with STX17 and UVRAG showed better predictive potential. These five genes, and in particular SLC7A11, may represent promising therapeutic targets and potential biomarkers in OC.

## Data Availability Statement

The raw data supporting the conclusions of this article will be made available by the authors, without undue reservation.

## Ethics Statement

The studies involving human participants were reviewed and approved by Ethical Review Committee, Guangxi Medical University. Written informed consent for participation was not required for this study in accordance with the national legislation and the institutional requirements.

## Author Contributions

YK, XC and YS performed most of the experiments. YK and FY wrote the manuscript. CC, SL, LX, DW, HZ, and CD performed the bioinformatics analysis. XL helped draft the manuscript. XL and FY designed and reviewed the study. All authors contributed to the article and approved the submitted version.

## Funding

This work was supported by the National Natural Science Foundation of China (81860458, 81903644, 81660606), Guangxi Natural Science Foundation (2021GXNSFAA075002, 2018GXNSFAA281227), and the Program Foundation of the Key Laboratory of High-Incidence Tumor Prevention and Treatment, Ministry of Education (GKE2019-20, GKE-ZZ202017, GKE-ZZ202148).

## Conflict of Interest

The authors declare that the research was conducted in the absence of any commercial or financial relationships that could be construed as a potential conflict of interest.

## Publisher’s Note

All claims expressed in this article are solely those of the authors and do not necessarily represent those of their affiliated organizations, or those of the publisher, the editors and the reviewers. Any product that may be evaluated in this article, or claim that may be made by its manufacturer, is not guaranteed or endorsed by the publisher.

## References

[1] Cho KR Shih Ie M. Ovarian Cancer. Annu Rev Pathol (2009) 4:287–313. doi: 10.1146/annurev.pathol.4.110807.092246 18842102 PMC2679364

[2] Stewart C Ralyea C Lockwood S. Ovarian Cancer: An Integrated Review. Semin Oncol Nurs (2019) 35(2):151–6. doi: 10.1016/j.soncn.2019.02.001 30867104

[3] Armstrong DK Alvarez RD Bakkum-Gamez JN Barroilhet L Behbakht K Berchuck A. Ovarian Cancer, Version 2.2020, NCCN Clinical Practice Guidelines in Oncology. J Natl Compr Canc Netw (2021) 19(2):191–226. doi: 10.6004/jnccn.2021.0007 33545690

[4] Ma RQ Tang ZJ Ye X Cheng HY Sun KK Chang XH. Overexpression of GPNMB Predicts an Unfavorable Outcome of Epithelial Ovarian Cancer. Arch Gynecol Obstet (2018) 297(5):1235–44. doi: 10.1007/s00404-018-4699-3 29428978

[5] Gasiorkiewicz BM Koczurkiewicz-Adamczyk P Piska K Pekala E. Autophagy Modulating Agents as Chemosensitizers for Cisplatin Therapy in Cancer. Invest New Drugs (2021) 39(2):538–63. doi: 10.1007/s10637-020-01032-y PMC7960624 33159673

[6] Koppula P Zhuang L Gan B. Cystine Transporter SLC7A11/xCT in Cancer: Ferroptosis, Nutrient Dependency, and Cancer Therapy. Protein Cell (2020) 12(8):599–620. doi: 10.1007/s13238-020-00789-5 33000412 PMC8310547

[7] Drayton RM Dudziec E Peter S Bertz S Hartmann A Bryant HE. Reduced Expression of miRNA-27a Modulates Cisplatin Resistance in Bladder Cancer by Targeting the Cystine/Glutamate Exchanger SLC7A11. Clin Cancer Res (2014) 20(7):1990–2000. doi: 10.1158/1078-0432.CCR-13-2805 24516043 PMC3974662

[8] Ge C Cao B Feng D Zhou F Zhang J Yang N. The Down-Regulation of SLC7A11 Enhances ROS Induced P-Gp Over-Expression and Drug Resistance in MCF-7 Breast Cancer Cells. Sci Rep (2017) 7(1):3791. doi: 10.1038/s41598-017-03881-9 PMC5476638 28630426

[9] Koppula P Zhang Y Shi J Li W Gan B. The Glutamate/Cystine Antiporter SLC7A11/xCT Enhances Cancer Cell Dependency on Glucose by Exporting Glutamate. J Biol Chem (2017) 292(34):14240–9. doi: 10.1074/jbc.M117.798405 PMC5572906 28630042

[10] Luo Y Wang C Yong P Ye P Liu Z Fu Z. Decreased Expression of the Long Non-Coding RNA SLC7A11-AS1 Predicts Poor Prognosis and Promotes Tumor Growth in Gastric Cancer. Oncotarget (2017) 8(68):112530–49. doi: 10.18632/oncotarget.22486 PMC5762530 29348845

[11] Hu K Li K Lv J Feng J Chen J Wu H. Suppression of the SLC7A11/glutathione Axis Causes Synthetic Lethality in KRAS-Mutant Lung Adenocarcinoma. J Clin Invest (2020) 130(4):1752–66. doi: 10.1172/JCI124049 PMC7108883 31874110

[12] Briggs KJ Koivunen P Cao S Backus KM Olenchock BA Patel H. Paracrine Induction of HIF by Glutamate in Breast Cancer: EglN1 Senses Cysteine. Cell (2016) 166(1):126–39. doi: 10.1016/j.cell.2016.05.042 PMC4930557 27368101

[13] Ma L Chen T Zhang X Miao Y Tian X Yu K. The M(6)A Reader YTHDC2 Inhibits Lung Adenocarcinoma Tumorigenesis by Suppressing SLC7A11-Dependent Antioxidant Function. Redox Biol (2021) 38:101801. doi: 10.1016/j.redox.2020.101801 33232910 PMC7691619

[14] Guan Z Chen J Li X Dong N. Tanshinone IIA Induces Ferroptosis in Gastric Cancer Cells Through P53-Mediated SLC7A11 Down-Regulation. Biosci Rep (2020) 40(8):BSR20201807. doi: 10.1042/BSR20201807 32776119 PMC7953492

[15] Polewski MD Reveron-Thornton RF Cherryholmes GA Marinov GK Aboody KS. SLC7A11 Overexpression in Glioblastoma Is Associated With Increased Cancer Stem Cell-Like Properties. Stem Cells Dev (2017) 26(17):1236–46. doi: 10.1089/scd.2017.0123 PMC5576215 28610554

[16] Ji X Qian J Rahman SMJ Siska PJ Zou Y Harris BK. xCT (SLC7A11)-Mediated Metabolic Reprogramming Promotes Non-Small Cell Lung Cancer Progression. Oncogene (2018) 37(36):5007–19. doi: 10.1038/s41388-018-0307-z PMC6127081 29789716

[17] Lee JR Roh J-L Lee SM Park Y Cho K-J Choi S-H. Overexpression of Cysteine-Glutamate Transporter and CD44 for Prediction of Recurrence and Survival in Patients With Oral Cavity Squamous Cell Carcinoma. Head Neck (2018) 40(11):2340–6. doi: 10.1002/hed.25331 30303590

[18] Shin SS Jeong BS Wall BA Li J Shan NL Wen Y. Participation of xCT in Melanoma Cell Proliferation In Vitro and Tumorigenesis *In Vivo* . Oncogenesis (2018) 7(11):86. doi: 10.1038/s41389-018-0098-7 30425240 PMC6234219

[19] Shiozaki A Iitaka D Ichikawa D Nakashima S Fujiwara H Okamoto K. xCT, Component of Cysteine/Glutamate Transporter, as an Independent Prognostic Factor in Human Esophageal Squamous Cell Carcinoma. J Gastroenterol (2014) 49(5):853–63. doi: 10.1007/s00535-013-0847-5 23771433

[20] Yoshikawa M Tsuchihashi K Ishimoto T Yae T Motohara T Sugihara E. xCT Inhibition Depletes CD44v-Expressing Tumor Cells That are Resistant to EGFR-Targeted Therapy in Head and Neck Squamous Cell Carcinoma. Cancer Res (2013) 73(6):1855–66. doi: 10.1158/0008-5472.CAN-12-3609-T 23319806

[21] Sun D Li YC Zhang XY. Lidocaine Promoted Ferroptosis by Targeting miR-382-5p /SLC7A11 Axis in Ovarian and Breast Cancer. Front Pharmacol (2021) 12:681223. doi: 10.3389/fphar.2021.681223 34122108 PMC8188239

[22] Mukhopadhyay S Biancur DE Parker SJ Yamamoto K Banh RS Paulo JA. Autophagy Is Required for Proper Cysteine Homeostasis in Pancreatic Cancer Through Regulation of SLC7A11. Proc Natl Acad Sci USA (2021) 118(6):e2021475118. doi: 10.1073/pnas.2021475118 33531365 PMC8017731

[23] Wang Y Xiong H Liu D Hill C Ertay A Li J. Autophagy Inhibition Specifically Promotes Epithelial-Mesenchymal Transition and Invasion in RAS-Mutated Cancer Cells. Autophagy (2019) 15(5):886–99. doi: 10.1080/15548627.2019.1569912 PMC6517269 30782064

[24] Das CK Banerjee I Mandal M. Pro-Survival Autophagy: An Emerging Candidate of Tumor Progression Through Maintaining Hallmarks of Cancer. Semin Cancer Biol (2020) 66:59–74. doi: 10.1016/j.semcancer.2019.08.020 31430557

[25] Xia H Li S Li X Wang W Bian Y Wei S. Autophagic Adaptation to Oxidative Stress Alters Peritoneal Residential Macrophage Survival and Ovarian Cancer Metastasis. JCI Insight (2020) 5(18):e141115. doi: 10.1172/jci.insight.141115 PMC7526547 32780724

[26] Zheng X Chen W Hou H Li J Li H Sun X. Ginsenoside 20(S)-Rg3 Induced Autophagy to Inhibit Migration and Invasion of Ovarian Cancer. BioMed Pharmacother (2017) 85:620–6. doi: 10.1016/j.biopha.2016.11.072 27899249

[27] Yu JL Gao X. MicroRNA 1301 Inhibits Cisplatin Resistance in Human Ovarian Cancer Cells by Regulating EMT and Autophagy. Eur Rev Med Pharmacol Sci (2020) 24(4):1688–96. doi: 10.26355/eurrev_202002_20343 32141535

[28] Tay Y Kats L Salmena L Weiss D Tan SM Ala U. Coding-Independent Regulation of the Tumor Suppressor PTEN by Competing Endogenous mRNAs. Cell (2011) 147(2):344–57. doi: 10.1016/j.cell.2011.09.029 PMC3235920 22000013

[29] Cazalla D Yario T Steitz JA. Down-Regulation of a Host microRNA by a Herpesvirus Saimiri Noncoding RNA. Science (2010) 328(5985):1563–6. doi: 10.1126/science.1187197 PMC3075239 20558719

[30] Jeyapalan Z Deng Z Shatseva T Fang L He C Yang BB. Expression of CD44 3'-Untranslated Region Regulates Endogenous microRNA Functions in Tumorigenesis and Angiogenesis. Nucleic Acids Res (2011) 39(8):3026–41. doi: 10.1093/nar/gkq1003 PMC3082902 21149267

[31] Qi X Zhang DH Wu N Xiao JH Wang X Ma W. ceRNA in Cancer: Possible Functions and Clinical Implications. J Med Genet (2015) 52(10):710–8. doi: 10.1136/jmedgenet-2015-103334 26358722

[32] Dong C Yin F Zhu D Cai X Chen C Liu X. NCALD Affects Drug Resistance and Prognosis by Acting as a ceRNA of CX3CL1 in Ovarian Cancer. J Cell Biochem (2020) 121(11):4470–83. doi: 10.1002/jcb.29670 32030795

[33] Yang G Xiao X Rosen DG Cheng X Wu X Chang B. The Biphasic Role of NF-kappaB in Progression and Chemoresistance of Ovarian Cancer. Clin Cancer Res (2011) 17(8):2181–94. doi: 10.1158/1078-0432.CCR-10-3265 PMC3152795 21339307

[34] Schmittgen TD Livak KJ. Analyzing Real-Time PCR Data by the Comparative C(T) Method. Nat Protoc (2008) 3(6):1101–8. doi: 10.1038/nprot.2008.73 18546601

[35] Rao X Huang X Zhou Z Lin X. An Improvement of the 2ˆ(-Delta Delta CT) Method for Quantitative Real-Time Polymerase Chain Reaction Data Analysis. Biostat Bioinforma Biomath (2013) 3(3):71–85. 25558171 PMC4280562

[36] Homma K Suzuki K Sugawara H. The Autophagy Database: An All-Inclusive Information Resource on Autophagy That Provides Nourishment for Research. Nucleic Acids Res (2010) 39(Database):D986–90. doi: 10.1093/nar/gkq995 PMC3013813 20972215

[37] Deng W Ma L Zhang Y Zhou J Wang Y Liu Z. THANATOS: An Integrative Data Resource of Proteins and Post-Translational Modifications in the Regulation of Autophagy. Autophagy (2018) 14(2):296–310. doi: 10.1080/15548627.2017.1402990 29157087 PMC5902229

[38] de Leeuw N Dijkhuizen T Hehir-Kwa JY Carter NP Feuk L Firth HV. Diagnostic Interpretation of Array Data Using Public Databases and Internet Sources. Hum Mutat (2012) 33(6):930–40. doi: 10.1002/humu.22049 PMC5027376 26285306

[39] Cancer Genome Atlas Research, N. Integrated Genomic Analyses of Ovarian Carcinoma. Nature (2011) 474(7353):609–15. doi: 10.1038/nature10166 PMC3163504 21720365

[40] Cerami E Gao J Dogrusoz U Gross BE Sumer SO Aksoy BA. The Cbio Cancer Genomics Portal: An Open Platform for Exploring Multidimensional Cancer Genomics Data. Cancer Discov (2012) 2(5):401–4. doi: 10.1158/2159-8290.CD-12-0095 PMC3956037 22588877

[41] Gao J Aksoy BA Dogrusoz U Dresdner G Gross B Sumer SO. Integrative Analysis of Complex Cancer Genomics and Clinical Profiles Using the Cbioportal. Sci Signal (2013) 6(269):pl1. doi: 10.1126/scisignal.2004088 23550210 PMC4160307

[42] Gyorffy B Lanczky A Szallasi Z. Implementing an Online Tool for Genome-Wide Validation of Survival-Associated Biomarkers in Ovarian-Cancer Using Microarray Data From 1287 Patients. Endocr Relat Cancer (2012) 19(2):197–208. doi: 10.1530/ERC-11-0329 22277193

[43] Warde-Farley D Donaldson SL Comes O Zuberi K Badrawi R Chao P. The GeneMANIA Prediction Server: Biological Network Integration for Gene Prioritization and Predicting Gene Function. Nucleic Acids Res (2010) 38(Web Server issue):W214–220. doi: 10.1093/nar/gkq537 PMC2896186 20576703

[44] Li JH Liu S Zhou H Qu LH Yang JH. Starbase V2.0: Decoding miRNA-ceRNA, miRNA-ncRNA and Protein-RNA Interaction Networks From Large-Scale CLIP-Seq Data. Nucleic Acids Res (2014) 42(Database issue):D92–97. doi: 10.1093/nar/gkt1248 PMC3964941 24297251

[45] Sumazin P Yang X Chiu HS Chung WJ Iyer A Llobet-Navas D. An Extensive microRNA-Mediated Network of RNA-RNA Interactions Regulates Established Oncogenic Pathways in Glioblastoma. Cell (2011) 147(2):370–81. doi: 10.1016/j.cell.2011.09.041 PMC3214599 22000015

[46] Shannon P Markiel A Ozier O Baliga NS Wang JT Ramage D. Cytoscape: A Software Environment for Integrated Models of Biomolecular Interaction Networks. Genome Res (2003) 13(11):2498–504. doi: 10.1101/gr.1239303 PMC403769 14597658

[47] Kanehisa M Furumichi M Tanabe M Sato Y Morishima K. KEGG: New Perspectives on Genomes, Pathways, Diseases and Drugs. Nucleic Acids Res (2017) 45(D1):D353–61. doi: 10.1093/nar/gkw1092 PMC5210567 27899662

[48] Shi C Wang M. LINC01118 Modulates Paclitaxel Resistance of Epithelial Ovarian Cancer by Regulating miR-134/Abcc1. Med Sci Monit (2018) 24:8831–9. doi: 10.12659/MSM.910932 PMC6292151 30521500

[49] Wang JM Liu BQ Zhang Q Hao L Li C Yan J. ISG15 Suppresses Translation of ABCC2 via ISGylation of Hnrnpa2b1 and Enhances Drug Sensitivity in Cisplatin Resistant Ovarian Cancer Cells. Biochim Biophys Acta Mol Cell Res (2020) 1867(4):118647. doi: 10.1016/j.bbamcr.2020.118647 31926942

[50] Zhou H-H Chen X Cai L-Y Nan X-W Chen J-H Chen X-X. Erastin Reverses ABCB1-Mediated Docetaxel Resistance in Ovarian Cancer. Front Oncol (2019) 9:1398. doi: 10.3389/fonc.2019.01398 31921655 PMC6930896

[51] Martincuks A Li PC Zhao Q Zhang C Li YJ Yu H. CD44 in Ovarian Cancer Progression and Therapy Resistance-A Critical Role for STAT3. Front Oncol (2020) 10:589601. doi: 10.3389/fonc.2020.589601 33335857 PMC7736609

[52] Uddin MH Kim B Cho U Azmi AS Song YS. Association of ALDH1A1-NEK-2 Axis in Cisplatin Resistance in Ovarian Cancer Cells. Heliyon (2020) 6(11):e05442. doi: 10.1016/j.heliyon.2020.e05442 33241139 PMC7672295

[53] Li X Zou Z Tang J Zheng Y Liu Y Luo Y. NOS1 Upregulates ABCG2 Expression Contributing to DDP Chemoresistance in Ovarian Cancer Cells. Oncol Lett (2019) 17(2):1595–602. doi: 10.3892/ol.2018.9787 PMC6341833 30675218

[54] Roy L Samyesudhas SJ Carrasco M Li J Joseph S Dahl R. ARID3B Increases Ovarian Tumor Burden and Is Associated With a Cancer Stem Cell Gene Signature. Oncotarget (2014) 5(18):8355–66. doi: 10.18632/oncotarget.2247 PMC4226688 25327563

[55] Guo H Ha C Dong H Yang Z Ma Y Ding Y. Cancer-Associated Fibroblast-Derived Exosomal microRNA-98-5p Promotes Cisplatin Resistance in Ovarian Cancer by Targeting CDKN1A. Cancer Cell Int (2019) 19:347. doi: 10.1186/s12935-019-1051-3 31889899 PMC6925473

[56] Mrkvicova A Chmelarova M Peterova E Havelek R Baranova I Kazimirova P. The Effect of Sodium Butyrate and Cisplatin on Expression of EMT Markers. PloS One (2019) 14(1):e0210889. doi: 10.1371/journal.pone.0210889 30653577 PMC6336326

[57] Zhang Y Tao L Fan LX Huang K Luo HM Ge H. Cx32 Mediates Cisplatin Resistance in Human Ovarian Cancer Cells by Affecting Drug Efflux Transporter Expression and Activating the EGFRAkt Pathway. Mol Med Rep (2019) 19(3):2287–96. doi: 10.3892/mmr.2019.9876 30664215

[58] D'Andrea AD. Mechanisms of PARP Inhibitor Sensitivity and Resistance. DNA Repair (Amst) (2018) 71:172–6. doi: 10.1016/j.dnarep.2018.08.021 30177437

[59] Stover EH Baco MB Cohen O Li YY Christie EL Bagul M. Pooled Genomic Screens Identify Anti-Apoptotic Genes as Targetable Mediators of Chemotherapy Resistance in Ovarian Cancer. Mol Cancer Res (2019) 17(11):2281–93. doi: 10.1158/1541-7786.MCR-18-1243 PMC6825578 31462500

[60] Mancini F Di Conza G Pellegrino M Rinaldo C Prodosmo A Giglio S. MDM4 (MDMX) Localizes at the Mitochondria and Facilitates the P53-Mediated Intrinsic-Apoptotic Pathway. EMBO J (2009) 28(13):1926–39. doi: 10.1038/emboj.2009.154 PMC2711189 19521340

[61] Deng J Bai X Feng X Ni J Beretov J Graham P. Inhibition of PI3K/Akt/mTOR Signaling Pathway Alleviates Ovarian Cancer Chemoresistance Through Reversing Epithelial-Mesenchymal Transition and Decreasing Cancer Stem Cell Marker Expression. BMC Cancer (2019) 19(1):618. doi: 10.1186/s12885-019-5824-9 31234823 PMC6591840

[62] Zhao L Huang L Zhang J Fan J He F Zhao X. The Inhibition of BRAF Activity Sensitizes Chemoresistant Human Ovarian Cancer Cells to Paclitaxel-Induced Cytotoxicity and Tumor Growth Inhibition. Am J Transl Res (2020) 12(12):8084–98. PMC7791515 33437383

[63] Zhang Z Dou X Yang H Jia L Qin K Gao X. Association of Expression of P53, Livin, ERCC1, BRCA1 and PARP1 in Epithelial Ovarian Cancer Tissue With Drug Resistance and Prognosis. Pathol Res Pract (2020) 216(2):152794. doi: 10.1016/j.prp.2019.152794 31902551

[64] Szaflarski W Sujka-Kordowska P Pula B Jaszczynska-Nowinka K Andrzejewska M Zawierucha P. Expression Profiles of Vault Components MVP, TEP1 and vPARP and Their Correlation to Other Multidrug Resistance Proteins in Ovarian Cancer. Int J Oncol (2013) 43(2):513–20. doi: 10.3892/ijo.2013.1975 23739867

[65] Wang Z. ErbB Receptors and Cancer. Methods Mol Biol (2017) 1652:3–35. doi: 10.1007/978-1-4939-7219-7_1 28791631

[66] Collot T Niogret J Carnet M Chevrier S Humblin E Favier L. PARP Inhibitor Resistance and TP53 Mutations in Patients Treated With Olaparib for BRCA-Mutated Cancer: Four Case Reports. Mol Med Rep (2021) 23(1):75. doi: 10.3892/mmr.2020.11713 33236159

[67] Xiao X Melton DW Gourley C. Mismatch Repair Deficiency in Ovarian Cancer — Molecular Characteristics and Clinical Implications. Gynecol Oncol (2014) 132(2):506–12. doi: 10.1016/j.ygyno.2013.12.003 24333356

[68] Lui GYL Shaw R Schaub FX Stork IN Gurley KE Bridgwater C. BET, SRC, and BCL2 Family Inhibitors are Synergistic Drug Combinations With PARP Inhibitors in Ovarian Cancer. EBioMedicine (2020) 60:102988. doi: 10.1016/j.ebiom.2020.102988 32927276 PMC7494677

[69] Ju X Yu H Liang D Jiang T Liu Y Chen L. LDR Reverses DDP Resistance in Ovarian Cancer Cells by Affecting ERCC-1, Bcl-2, Survivin and Caspase-3 Expressions. Biomed Pharmacother (2018) 102:549–54. doi: 10.1016/j.biopha.2018.03.092 29597088

[70] Jin P Liu Y Wang R. STAT3 Regulated miR-216a Promotes Ovarian Cancer Proliferation and Cisplatin Resistance. Biosci Rep (2018) 38(4):BSR20180547. doi: 10.1042/BSR20180547 30061175 PMC6131203

[71] Zhong W Weiss HL Jayswal RD Hensley PJ Downes LM St. Clair DK. Extracellular Redox State Shift: A Novel Approach to Target Prostate Cancer Invasion. Free Radical Biol Med (2018) 117:99–109. doi: 10.1016/j.freeradbiomed.2018.01.023 29421238 PMC5845758

[72] Timmerman LA Holton T Yuneva M Louie RJ Padro M Daemen A. Glutamine Sensitivity Analysis Identifies the xCT Antiporter as a Common Triple-Negative Breast Tumor Therapeutic Target. Cancer Cell (2013) 24(4):450–65. doi: 10.1016/j.ccr.2013.08.020 PMC3931310 24094812

[73] Lanzardo S Conti L Rooke R Ruiu R Accart N Bolli E. Immunotargeting of Antigen xCT Attenuates Stem-Like Cell Behavior and Metastatic Progression in Breast Cancer. Cancer Res (2016) 76(1):62–72. doi: 10.1158/0008-5472.CAN-15-1208 26567138

[74] Sugano K Maeda K Ohtani H Nagahara H Shibutani M Hirakawa K. Expression of xCT as a Predictor of Disease Recurrence in Patients With Colorectal Cancer. Anticancer Res (2015) 35(2):677–82. 25667445

[75] Sato M Kusumi R Hamashima S Kobayashi S Sasaki S Komiyama Y. The Ferroptosis Inducer Erastin Irreversibly Inhibits System Xc- and Synergizes With Cisplatin to Increase Cisplatin's Cytotoxicity in Cancer Cells. Sci Rep (2018) 8(1):968. doi: 10.1038/s41598-018-19213-4 29343855 PMC5772355

[76] Ma MZ Chen G Wang P Lu WH Zhu CF Song M. Xc- Inhibitor Sulfasalazine Sensitizes Colorectal Cancer to Cisplatin by a GSH-Dependent Mechanism. Cancer Lett (2015) 368(1):88–96. doi: 10.1016/j.canlet.2015.07.031 26254540

[77] Guo W Zhao Y Zhang Z Tan N Zhao F Ge C. Disruption of xCT Inhibits Cell Growth via the ROS/autophagy Pathway in Hepatocellular Carcinoma. Cancer Lett (2011) 312(1):55–61. doi: 10.1016/j.canlet.2011.07.024 21906871

[78] Muinao T Deka Boruah HP Pal M. Diagnostic and Prognostic Biomarkers in Ovarian Cancer and the Potential Roles of Cancer Stem Cells – An Updated Review. Exp Cell Res (2018) 362(1):1–10. doi: 10.1016/j.yexcr.2017.10.018 29079264

[79] Sheng R Li X Wang Z Wang X. Circular RNAs and Their Emerging Roles as Diagnostic and Prognostic Biomarkers in Ovarian Cancer. Cancer Lett (2020) 473:139–47. doi: 10.1016/j.canlet.2019.12.043 31904484

[80] Alharbi M Zuñiga F Elfeky O Guanzon D Lai A Rice GE. The Potential Role of miRNAs and Exosomes in Chemotherapy in Ovarian Cancer. Endocr Relat Cancer (2018) 25(12):R663–85. doi: 10.1530/erc-18-0019 30400025

[81] Yin F Yi S Wei L Zhao B Li J Cai X. Microarray-Based Identification of Genes Associated With Prognosis and Drug Resistance in Ovarian Cancer. J Cell Biochem (2018) 120(4):6057–70. doi: 10.1002/jcb.27892 30335894

[82] Wei Q-Y Meyer MF Seuthe IMC Drebber U Siefer O Kreppel M. Valosin-Containing Protein (VCP/p97)-Expression Correlates With Prognosis of HPV- Negative Oropharyngeal Squamous Cell Carcinoma (OSCC). PloS One (2014) 9(12):e114170. doi: 10.1371/journal.pone.0114170 25463965 PMC4252085

[83] Yun CW Jeon J Go G Lee JH Lee SH. The Dual Role of Autophagy in Cancer Development and a Therapeutic Strategy for Cancer by Targeting Autophagy. Int J Mol Sci (2020) 22(1):179. doi: 10.3390/ijms22010179 PMC7795059 33375363

[84] Silva VR Neves SP Santos LS Dias RB Bezerra DP. Challenges and Therapeutic Opportunities of Autophagy in Cancer Therapy. Cancers (Basel) (2020) 12(11):3461. doi: 10.3390/cancers12113461 PMC7699739 33233671

[85] Usman RM Razzaq F Akbar A Farooqui AA Iftikhar A Latif A. Role and Mechanism of Autophagy-Regulating Factors in Tumorigenesis and Drug Resistance. Asia Pac J Clin Oncol (2020) 17(3):193–208. doi: 10.1111/ajco.13449 32970929

[86] Qiu S Sun L Jin Y An Q Weng C Zheng J. Silencing of BAG3 Promotes the Sensitivity of Ovarian Cancer Cells to Cisplatin via Inhibition of Autophagy. Oncol Rep (2017) 38(1):309–16. doi: 10.3892/or.2017.5706 28628188

[87] Luan W Pang Y Li R Wei X Jiao X Shi J. Akt/mTOR-Mediated Autophagy Confers Resistance To BET Inhibitor JQ1 In Ovarian Cancer. Onco Targets Ther (2019) 12:8063–74. doi: 10.2147/OTT.S220267 PMC6781612 31632060

